# Advances in Lipid and Metal Nanoparticles for Antimicrobial Peptide Delivery

**DOI:** 10.3390/pharmaceutics11110588

**Published:** 2019-11-08

**Authors:** Marcin Makowski, Ítala C. Silva, Constança Pais do Amaral, Sónia Gonçalves, Nuno C. Santos

**Affiliations:** Instituto de Medicina Molecular, Faculdade de Medicina, Universidade de Lisboa, Av. Prof. Egas Moniz, 1649-028 Lisbon, Portugal; marcin.makowski@medicina.ulisboa.pt (M.M.); itala.silva@medicina.ulisboa.pt (Í.C.S.); maria.paisdoamaral@medicina.ulisboa.pt (C.P.d.A.)

**Keywords:** antimicrobial peptide, anticancer peptide, nanoparticle, metal nanoparticle, nanotoxicity, liposome

## Abstract

Antimicrobial peptides (AMPs) have been described as excellent candidates to overcome antibiotic resistance. Frequently, AMPs exhibit a wide therapeutic window, with low cytotoxicity and broad-spectrum antimicrobial activity against a variety of pathogens. In addition, some AMPs are also able to modulate the immune response, decreasing potential harmful effects such as sepsis. Despite these benefits, only a few formulations have successfully reached clinics. A common flaw in the druggability of AMPs is their poor pharmacokinetics, common to several peptide drugs, as they may be degraded by a myriad of proteases inside the organism. The combination of AMPs with carrier nanoparticles to improve delivery may enhance their half-life, decreasing the dosage and thus, reducing production costs and eventual toxicity. Here, we present the most recent advances in lipid and metal nanodevices for AMP delivery, with a special focus on metal nanoparticles and liposome formulations.

## 1. Introduction

The so-called post-antimicrobial era is dangerously approaching, as antimicrobial resistance (AMR) spreads due to the overuse and misuse of antibiotics. AMR is among the major threats to public health. In particular, the feasibility of many regular medical procedures such as the treatment of infections, surgeries, or intensive care medicine is dependent on the effectivity of antibiotics [1]. Projections estimate that AMR could be responsible for up to 10 million deaths per year by 2050, surpassing the mortality of cancer [2]. This scenario has pushed the search for new alternatives to fight bacterial infections.

Antimicrobial peptides (AMPs) have been pointed out as a potential new generation of antibiotic molecules to fight AMR [3]. These peptides are ancient innate immune effectors widely expressed in nature. AMPs are generally short (10–50 amino acid residues) cationic amphiphilic molecules that exhibit antibacterial activity, often through membrane permeabilization [4]. These peptides may present broad-spectrum activity, being effective against a variety of infectious bacteria, viruses, fungi and parasites, but also against tumors [5,6,7,8,9,10]. The main forces driving the selectivity of AMPs are the electrostatic interactions between the cationic peptides and anionic molecules in the surface of the pathogenic cells. Bacteria membranes, for instance, are richer in anionic phospholipids, when compared to a healthy eukaryotic cell. Cancer cells, on the other hand, frequently experience a higher exposure of anionic phospholipids, such as phosphatidylserines, on the outer leaflet of their plasma membrane. This feature enables the interaction of AMPs with cancer cells. After binding to the membranes, hydrophobic residues of AMPs promote their insertion in the bilayer. This may lead to membrane disruption through diverse mechanisms, including pore formation. Some AMPs can cross the membrane as well and interact with intracellular targets, namely by inhibiting nucleic acid, protein or cell-wall synthesis [11].

The selectivity of these peptides has been highlighted as an additional advantage of AMPs, displaying cytotoxic effects only at high concentrations, providing a wide therapeutic window [12]. In addition, some AMPs, such as the human LL-37, can act as signaling molecules exhibiting immunomodulatory, wound healing or angiomodulatory properties [13,14,15]. Their applications are also being investigated as promising antibiofilm drugs. Biofilms are bacteria communities that can attach to a variety of biotic and abiotic surfaces, including teeth, skin, vagina, prosthesis, catheters or stents [16]. They are recalcitrant due to the presence of dormant bacteria that are insensible to most antibiotics, which act strictly in metabolic active bacteria. Thus, biofilms can easily progress to chronic infections. Since the antimicrobial activity of AMPs is usually independent of the metabolic state of the targeting pathogen, they can eradicate even the most persistent dormant cells [17].

Decades of research have led to the discovery of more than 3000 AMPs, described and compiled in the AMP database (http://aps.unmc.edu/AP/). However, very few AMPs are already available to be used in clinics so far [12], and the number of AMPs that reach clinical trials is very low. Peptide drugs face additional problematics to the well-established difficulties in bringing new antibiotic drugs to the market [18]. AMPs frequently have low plasma stability, as they are susceptible to cleavage and inactivation by the proteases of host and pathogens, limiting the administration route [19,20]. Indeed, most AMPs that are being tested in clinical trials are restricted to topical use [12]. Furthermore, although the minimum inhibitory concentrations (MICs) of AMPs can be low, achieving those concentrations at the intended target is challenging. This increases the risk of undesired cytotoxicity. The unfavorable in vivo behavior of AMPs has also delayed their clinical implementation.

Searching all AMPs that are currently under clinical trials or that have been, in the clinical trial database (clinicaltriasl.gov), one can find 326 registered trials (Supplementary Data Table S1). Among those 326 trials, only 49 are currently running, while the remaining, even though in a completed state, have no published results yet. Only 54 from the total have published results. Considering the difficulties that emerge for AMP clinical application, updates about these trials should be published, even if the final results are not available yet. From the presented clinical trials, 40.8% have been updated within the last two years. Besides the few AMPs in clinical trials and the actual number of approved in clinics, the scientific community should make an effort to publish the results obtained and an investment on the development of better methods to overcome AMP in vivo instability.

Nanoparticles (NPs) are interesting drug delivery systems that may overcome current problems of drug design. NPs as drug delivery systems provide a way to protect AMPs from environmental challenges and to deliver them to the desired site, allowing thus to bypass the most common AMP limitations. Nanostructured materials can provide efficacy in specific targeting, controlled release, lower toxicity and better bioavailability [21]. Breakthroughs in the area of nanoengineering may overcome the drawbacks of AMPs and turn usable AMPs that were previously considered inadequate for clinical practice. We review here the recent advances on AMP delivery using lipid and metal nanoparticles. First, we will discuss NPs structural characterization and their influence on the peptide-NP conjugate biodistribution, followed by the peptide-NPs conjugation and their incorporation in nanostructured materials such as liposomes and metal nanoparticles. Then, we will address their application in therapeutics. Finally, we will provide a view of the most recent nanoparticle-based therapies and the current challenges for clinical translation.

Nanoparticles are colloidal nanomaterials used as drug delivery systems, among other purposes. In nanomedicine, NPs have sizes below 1000 nm [22]. They can be made out of a variety of materials such as lipids, metal colloids, polymers, dendrimers and hydrogels [23,24,25,26]. Nanomaterials possess singular properties that are reflected in their structural functionalities. Depending on the particle shape, size, surface area, charge and capping agent, they can interact with many biomolecules. These combinations have a critical role in their multiple functions, affecting their behavior [27]. To ensure a specific and stable biodistribution to the target site, it is necessary to assess their physicochemical characteristics. Different techniques are available, suitable for the peculiarities of each material [28].

Dynamic light scattering (DLS) is a widely used technique to assess the size distribution of colloidal systems. This technology is based on the measurement of the Brownian motion of particles by monitoring the light scattered by the colloidal particles at the µs timescale [29,30]. DLS can also measure the polydispersity of a sample, meaning how homogeneous the size of the colloidal suspension is. This is critical, as an homogeneous size population is required for an homogeneous biodistribution [31]. Zeta-potential measurements are a powerful tool to obtain an approximate value of the surface charge of a particle in suspension. To measure the zeta-potential, the electrophoretic mobility of the particles in suspension is monitored upon the application of an electrical field [29,30]. Zeta-potential is invaluable in nanomaterial engineering, as it is an indicator of the stability of the colloidal dispersion. For instance, zeta-potentials close to zero (as a rule of the thumb, between −30 and 30 mV) are indicative of a propensity for particle aggregation.

Immediately after contacting with the biological environment, NPs are covered by innumerous proteins. These proteins conform the ‘corona’, which can strongly influence the fate of a NP in vivo. For instance, protein corona can cause leakage from liposomes [32]. An important fraction of the blood serum proteins are opsonins. Opsonins are proteins of the innate immune system that act by marking an antigen for clearance by the mononuclear phagocytic system [33]. Phagocytic cells are mainly circulating in the bloodstream, but they are also found in the liver, spleen, lung and bone marrow [34]. These cells are responsible for the removal of pathogens, dead cells and cellular debris. Endocytosis depends on particle size [35,36]. NPs between 100 and 200 nm can accumulate in the liver and further be eliminated by Kupffer cells [37,38]. Larger NPs can accumulate and impregnate organs such as the liver, spleen and the bone marrow. NPs smaller than 100 nm are directed to the red pulp of the spleen and phagocytized by macrophages. NPs smaller than 5 nm are eliminated by renal clearance [39]. Liposomes are biodegradable molecules [40] and are easily eliminated from the organism, in contrast with metal nanoparticles that are durable materials and may accumulate in tissues, raising concerns for long-term toxicity and safety [39].

To avoid opsonization and phagocytic clearance, NPs can be modified by decoration with a variety of molecules to change the properties of the particle surface, postponing opsonization by the immune system. In addition to the use of peptides as capping agent, the most common molecules used for this purpose are polyethylene glycol (PEG), chitosan, citrate, polyvinyl alcohol (PVA) and polyvinylpyrrolidone (PVP) [41,42,43]. In addition to this function, the use of capping agents is important in the delivery of hydrophobic coating NPs (which tend to clump together and are readily captured by phagocytes from the mononuclear system). When coated with hydrophilic polymers, NPs become easier to deliver to their target [44].

AMPs are produced by the innate immune system. Some of them are also known as host defense peptides (HDPs) and have an immunomodulatory function, being in the front line of host defense against infections. They are located in the granules of phagocytic cells or induced in epithelial cells during inflammatory response [45]. AMPs can be secreted extracellularly in the site of inflammation and exert their antimicrobial effects [46,47]. The use of NPs can protect AMPs from being recognized by the immune system and ensure the adequate dose delivery for their specific target. For in vivo application, NPs must avoid the immune system detection, without being immunotoxic or inhibiting the immune response homeostasis [48]. To achieve these features, NPs’ sterility and lipopolysaccharide (LPS or endotoxin) contamination must be evaluated for each NP batch. Understanding the importance of NPs’ immunocompatibility is essential to develop strategies to search for NPs-mediated immune reactions [39,49]. Crist et al. [49] demonstrated how residual synthesis byproducts act as immunoreactive components and can mask real NPs’ cytotoxic effects. Contamination with bacteria LPS may activate monocytes and dendritic cells (DCs), causing immunostimulatory reactions that that may initiate an overstimulation of the immune system, which may lead to a strong inflammatory response and even to the life-threatening septic syndrome [50].

## 2. Lipid-Based Nanoparticles

Lipid nanoparticles (LNPs) are promising drug delivery systems (DDSs). An obvious advantage over other synthetic formulations is the safety and biocompatibility of the materials they are made of. In addition, lipid-based drug delivery systems are exceptionally versatile, being able to transport hydrophobic and hydrophilic molecules. This factor makes them especially attractive choices for AMPs delivery. Indeed, LNPs have been investigated as vehicles for peptide drugs for more than three decades [51].

LNPs are commonly used for dermatological pathologies, as they can reach deeper layers of skin and such conditions need topical action [52]. In general, topical application of drugs is a safer method of drug delivery, due to reduced side effects and effective action [53]. As for other routes of administration, recently, a new formulation of lipid nanoparticles for intranasal treatment of Parkinson disease using the glial-derived growth neurotrophic factor (GDNF) encapsulated in a chitosan (CS)-coated nanostructured lipid carriers (NLC), CS-NLC-TAT-GDNF, is in progress and has showed better brain delivery when compared to previous formulations [54]. Similar results were observed using curcumin lipid nanocarriers through intranasal administration in in vitro models of Alzheimer’s disease [55]. Alongside these routes of administration, systemic and oral are the most common. Intravenous administration is still a very frequent method, due to a more accurate drug distribution to the target site and reduced therapeutic agent degradation, when compared, for instance, to oral administration [56].

### 2.1. Liposomes

**Figure 1:**
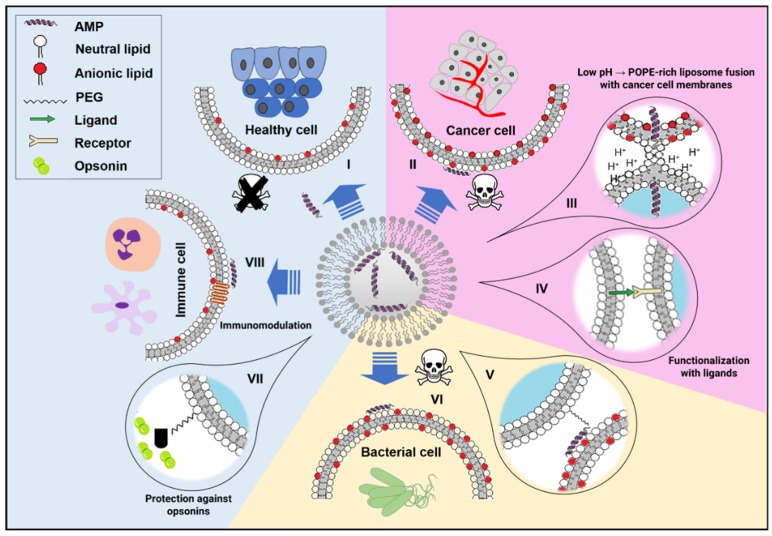
Strategies to deliver antimicrobial peptides (AMPs) using liposomes. Liposomes can be decorated and their lipid composition tuned to optimize their delivery capacities. Frequently, the delivery of AMPs is achieved through diffusion, here represented by the blue arrows. From top left and clockwise: (**I**) Healthy host cells have decreased susceptibility to AMPs, as their surface is close to zwitterionic (almost neutral net charge), which prevents the cationic AMPs form interacting with them. (**II**) The plasma membranes of cancer cells lose the natural phospholipid asymmetry between the two membrane leaflets, with the anionic phospholipids, which are usually concentrated on the inner monolayer of the membrane, becoming exposed on the outer leaflet and, therefore, promoting the interaction of the cationic AMPs with the anionic surface of the cancer cell. (**III**) Extracellular acidification is also a signature feature of cancer cells. Lipid nanoparticles enriched in POPE have a propensity to form non-lamellar phases at low pH, turning the lipid nanoparticles fusogenic, which can be used as strategy to deliver a cargo to these cells. (**IV**) Lipid nanoparticles can be functionalized with ligands that bind to receptors differentially expressed by cancer cells. (**V**) The surface of lipid nanoparticles can be functionalized PEGylated AMPs for a direct action of the peptide on the membrane of the target cell. (**VI**) Bacteria have anionic phospholipids and/or other anionic biomolecules exposed on their surface, favoring the interaction with AMPs. (**VII**) The surface of stealth lipid nanoparticles is decorated with polymers that prevent the formation of a protein corona and opsonization. (**VIII**) Some AMPs have immunomodulatory properties that can prevent potentially dangerous inflammatory over-reactions.

Liposomes are the most well-known, most used and easy to produce LNP system for drug administration, being that they were the first nanomedicine approved by the regulatory agencies [57]. They are spherical self-enclosed lipid bilayers that can be spontaneously formed in aqueous suspension. The main component of liposomes are phospholipids, although other lipid species, namely cholesterol (Chol), are also frequently incorporated. They are biodegradable, biocompatible and show very low toxicity and immunogenicity [58]. The use of liposomes as drug carriers was proposed during the 1970s by Gregory Gregoriadis [59]. Two decades later, the anti-cancer PEGylated liposomal doxorubicin (Doxil^®^) was the first nanodrug approved by the United States Food and Drug Administration (FDA) [57]. Liposome formulations are currently the leading nanocarrier platform in medicine [60]. They are particularly suited to deliver amphiphilic peptides, such as AMPs, as upon encapsulation AMPs remain protected of proteolytical degradation by the phospholipid bilayer. Some of the strategies used to deliver AMPs using liposomes are illustrated in Figure 1.

There is no liposome preparation ‘golden rule’. Depending on the intended use, their composition, size and surface modifications should be tuned. The most widely used method for liposome preparation is the ‘thin film hydration’ followed by extrusion or sonication to narrow down the size distribution. However, these methods often involve the use of organic solvents to handle the lipids, which makes them unsuitable for pharmaceutical application. A variety of methods that do not rely on the use of toxic organic solvents have been patented, while many other are under development. Koyonova and Tenchov have recently reviewed the trends on liposome production for pharmaceutical purposes [61].

The selection of a lipid composition suitable for the delivery of a given AMP is of paramount importance. Lipid composition will determine the physical properties of the liposome, including packing and charge. Lipid packing and membrane fluidity will influence AMP encapsulation efficiency and stability of the liposomes [62]. The main factor affecting packing and fluidity is the degree of unsaturation of the phospholipids. Fluidity increases with the unsaturation degree. Cholesterol, in turn, favors the stabilization of the packing of the bilayers, and is thus beneficial for liposome half-life [63]. On the other hand, adding Chol can be detrimental for AMP encapsulation efficiency [64].

Phosphatidylethanolamines (PE) have intrinsic negative curvature and are thus not prone to form lamellar phases. The addition of PE may lower the packing, being thus detrimental for stability, but is often selected for the design of fusogenic liposomes that can fuse to the target cell membranes and deliver their cargo into the cytoplasm. Indeed, liposomes encapsulating AMPs targeting a tumor microenvironment frequently take advantage of this feature. Under the low pH associated with the tumor microenvironment, PE headgroups can be protonated, resulting in a major destabilization of the liposome, triggering the release of the AMP to the tumor (Figure 1) [65,66]. Targeting specifically tumor acidosis is particularly interesting as pH dysregulation *per se* can cause resistance to chemotherapy and immunotherapy [67]. Anionic phospholipids, such as phosphatidylglycerols (PG), are generally avoided in the preparation of AMP-liposome carriers. The most obvious reason for this is that many cationic AMPs exert their membrane disrupting activity only in the presence of anionic phospholipids. That is the basis for the safety of AMPs. Thus, adding PG to the liposomes that carry AMPs may end in the complete disruption of the expected carrier of the peptide. However, the AMP nisin (net charge +4) is inactive when encapsulated in uncharged liposomes, but shows high antimicrobial activity when encapsulated in PG-containing liposomes [68]. One could ask why nisin does not disrupt the anionic phospholipid bilayer that encloses it. It has been suggested that the explanation to this is the high affinity of nisin to lipid II, a lipid that participates in the synthesis of the peptidoglycan cell wall in many bacteria. Without the interaction with lipid II, nisin does not form pores in membranes [69]. As a final remark on the lipid composition, a general rule for pharmaceutical purposes is ‘the simpler, the better’, as complex formulations or formulations with complex coatings require extra pharmacokinetic and pharmacodynamic studies [70].

Understanding the interactions of the liposomal formulations with the diseased environment is crucial for the success of the formulation. Alipour et al. [71] observed that the polyanions present in the sputum of cystic fibrosis patients affected the antimicrobial activity of naked polymyxin B (PB), due to electrostatic neutralization. This was prevented with the liposomal PB [71]. In this regard, He et al. [72] showed that intravenous injection of liposomal PB improved the serum pharmacokinetic profile of PB in mice. Moreover, liposomal PB was more effectively targeted to the site of infection than the naked form. Li et al. [73] studied the pharmacokinetics and pharmacodynamics of liposomal-encapsulated daptomycin against *Staphylococcus aureus* in skin infection models. The liposomal formulation, ‘flexible-nanoliposomes’, based on a mixture of lecithin and sodium cholate, was able to permeate the skin efficiently, inhibiting bacteria growth across the tissues within the skin.

As previously mentioned, charged liposomes have an enhanced propensity to interact with serum proteins such as opsonins that will mark the liposome for phagocytic clearance. Associated with the opsonization by complement proteins, some liposome therapy patients can develop an acute syndrome known as complement activation-related pseudoallergy [74]. In the manufacturing of liposomes, it is very frequent to use surface modifications with ‘stealth’ materials such as PEG. These moieties will act as a steric barrier against the adhesion of opsonins (Figure 1). However, the voracity of phagocytes for liposomes has also been used as an advantage in cases where these cells are the therapeutic target. Indeed, many pathogenic bacteria have evolved to escape phagosomal degradation through several mechanisms (which has been named the macrophage paradox [75]). These bacteria can survive and replicate in diverse compartments inside the macrophage. Pathogenic bacteria able to replicate in macrophages include *Legionella pneumophila*, *Listeria monocytogenes*, *Mycobacterium tuberculosis*, *Francisella tularensis*, *Salmonella enterica* and *Chlamydia pneumonia* [76]. Liposomes have been used as a Trojan horse to deliver antibiotics to kill intracellular pathogens affecting macrophages [77,78]. This ‘Trojan horse’ strategy, however, has not been explored with AMPs. A possible explanation to this is that intracellularly AMPs may interfere with mitochondrial activity, which can trigger apoptotic death of the cell [79].

The size of liposomes used in nanomedicine varies from 50 to 500 nm, depending on the purpose [80]. It has been noticed that liposomes smaller than 200 nm may passively accumulate at the target site. This phenomenon, named enhanced permeability and retention (EPR), is pivotal for many liposome-based therapies [81,82]. EPR is caused by an increased local leakiness of the endothelial cells of the vessels, which occurs in several pathologies, including infection and cancer, due to inflammation.

#### 2.1.1. Liposomal Antimicrobial Peptide (AMP) Formulations against Bacteria Infections

Polymyxin B, an antimicrobial lipopeptide, was responsible for the first success story of an anti-infectious liposomal formulation. Polymyxins were discovered in the 1940s, but their clinical use declined in the 1970s due to their nephrotoxicity [83,84]. The first attempts to encapsulate PB in liposomes were done in the 1990s. Early studies showed that PB encapsulation in charged liposomes was not detrimental to its antimicrobial activit y [85,86].

Liposomal bacteriocin formulations aided to fight a series of *L. monocytogenes* outbreaks in the late 1990s and 2000s [68,87,88]. Bacteriocins are ribosomally synthetized bactericidal peptides. Their production can be a determinant for a bacterium to outcompete other bacteria when colonizing a new environment [89]. As other AMPs, bacteriocins suffered a delay in their clinical implementation, mainly due to undesirable cytotoxic effects. Antimicrobial resistance development has also been a problem associated with these peptides. Nonetheless, several bacteriocins are widely used in the food industry as food preservatives [90]. A strategy to overcome nisin resistance studied by Pinilla et al. [91] consists in the co-encapsulation of nisin with garlic extract. They studied the activity of this combination against several frequent bacteria contaminants in the food industry, finding that this combination could fight both Gram-negative and Gram-positive food-borne pathogens. Liposome bacteriocins have been particularly useful to fight *L. monocytogenes* food contaminations. However, some bacteriocins have been shown to elicit resistance in *L. monocytogenes* upon the first treatment [92]. A strategy to avoid resistance acquisition in these treatments was developed by Malheiros et al. [93] using a mixture of bacteriocins from *Lactobacillus sakei* encapsulated in cationic liposomes, being able to delay listerial growth in goat ultra-high temperature processed (UHT) milk.

Synergistic effects have been gaining increasing attention, as they reduce the effective used dosage, which, in turn, may prevent selection of resistant strains, according to the mutant prevention concentration hypothesis [94]. Combining multispecies bacteriocins encapsulated in liposomes can be helpful to control infectious diseases. Sosunov et al. [95] achieved the inhibition of intracellular growth of *M. tuberculosis* in vivo using encapsulated bacteriocins from *Streptococcus cricetus*, *Lactobacillus salivarus* and *Enterococcus faecalis* in liposomes containing phosphatidylcholine (PC) and cardiolipin (CL). Li et al. [96] reported an effective anti-MRSA (multi-resistant *Staphylococcus aureus*) co-delivery of daptomycin and clarithromycin. The combination of the two antimicrobial molecules enabled the reduction of the dosage of the antimicrobial lipopeptide daptomycin, still obtaining a significantly increased in vivo survival rate of infected mice. Liposome co-encapsulation of synergist antimicrobials can result in improved in vivo safety. Intravenous administration of liposomes co-encapsulating an antimicrobial peptide called DP7 conjugated with cholesterol (DP7-CHOL) and azithromycin prevented the side effects associated to DP7 cytotoxicity and reduced MRSA counts [97]. It is also worth mentioning that the antimicrobial peptide fraction of this formulation apparently induced an immunomodulatory activity (Figure 1), reducing the expression of several pro-inflammatory cytokines and upregulating the anti-inflammatory ones, a desired effect to prevent sepsis.

Bacteria biofilms are multispecies communities immersed in a polymeric matrix that can adhere to a variety of biotic and abiotic surfaces. They are associated with a wide range of health care infections caused by medical devices used in patient treatment, such as catheters and prosthetic valves [16]. Biofilms are a hub for horizontal transfer genes, including those associated to drug-resistance, contributing to many persistent and chronic infections [98]. In addition, biofilm communities have a core of bacteria with low metabolic activity that are intrinsically resistant to many conventional antibiotics, making biofilms highly recalcitrant [99]. Furthermore, this core is far from reach for antibiotics and immune system mediators. Thus, the treatment of biofilms requires high doses of antimicrobial drugs, favoring resistance development [100]. Dental caries are a very common form of biofilm, frequently formed by a matrix of insoluble glucans produced by a community of streptococci. Glucan synthesis decreased with the concentration of nisin-loaded liposomes [101]. Moreover, the inclusion of the cationic lipid phytosphingosine in the liposomal formulation may improve the anticariogenic action [101].

From 136 liposome formulations combined with anti-infective drugs currently under clinical trials, only 20 are active, leaving the problem of little available clinical application of these delivery systems [102].

#### 2.1.2. Anticancer Liposomal AMPs

Several AMPs have also anticancer properties. Liposomes are convenient drug carriers to the tumor microenvironment, due to their EPR effect. Moreover, multidrug resistance is also a great concern in cancer treatment. It is thus not surprising that several attempts have been made to encapsulate AMPs in DDSs as an anticancer strategy. Liposomal co-encapsulation of the AMP chrysophsin-1, obtained from the red sea bream gills, with the anti-neoplasic drug epirubicin (Epi) increases the anticancer activity of Epi against HeLa cells [103]. The addition of Epi alone and with liposomes was prone to increase the expression of several multi-drug pumps. However, the co-encapsulation of Epi and chrysophsin-1 avoided that overexpression by a reactive oxygen species (ROS)-mediated inhibition mechanism [103]. In a similar study, a synergistic effect was observed between an iron metabolism-related AMP named hepcidin 2-3 and Epi when encapsulated in cationic liposomes [104]. The results of this study pointed out that the co-incubation of hepcidin 2-3 with Epi caused programmed cell death in cervical cancer through ROS-mediated disruption of several signaling pathways.

Melittin is an AMP present in the domestic bee venom. Despite its high antimicrobial activity, it is also hemolytic, which makes it unfit for intravenous administration [105]. Melittin modified poloxamer liposomes were studied against hepatic carcinoma in a mice xenograft tumor model. This formulation showed increased safety and anti-hepatocarcinoma activity in vivo, inducing apoptosis of the cancerous hepatic cells [106]. Melittin liposomes showed antitumor activity similar to the FDA-approved anticancer drug sorafenibin, reducing hepatic tumor size. Moreover, while naked melittin administration resulted in increased counts of neutrophils and eosinophils, indicating an inflammation and allergic reaction, melittin nano-liposomes effectively prevented anaphylaxis [106].

Photodynamic therapy (PDT) is a widely used methodology, particularly in cancer therapy [107]. PDT requires three ingredients: a highly photoreactive molecule (photosensitizer), a source of light and oxygen. Upon excitation with light, the photosensitizer will react with the oxygen molecules in the surroundings, leading to the generation of ROS. The ROS generated are extremely reactive and unspecific, and can oxidize lipids in the membranes, proteins and nucleic acids, leading to cytotoxicity [107]. Yang et al. [108] used an antimicrobial peptide to deliver liposome-encapsulated temoporfin, a photosensitizer, to *P. aeruginosa* and *S. aureus* cells.

The surface of liposomes can be modified so that they express ligands specific to a certain type of cell. This is very useful in anticancer drug delivery, as cancer cells frequently express receptors that other healthy cells do not. Ligands that can trigger receptor-mediated endocytosis are most desirable. Zhang et al. [109] dually functionalized a liposome with a pH responsive AMP and a α_v_β_3_ integrin-targeting ligand, showing efficient growth inhibition of tumors expressing that integrin. Nonetheless, in tumors with high genomic instability, phenotypic heterogeneity may arise, and it is possible that not all malignant cells express the receptor [110]. The receptor-independent mechanism of action of antimicrobial peptides is an advantageous feature to fight heterogeneous tumors.

The most frequently mentioned disadvantage of liposomes is their low stability in vivo (e.g., mononuclear phagocytic system clearance). Additionally, the lack of practical sterilization methods hampers the scaling-up of liposomal formulations [111,112]. Heat sterilization is inadequate for liposomes, as it would degrade the product. Chemical sterilization is also unfit, as chemical contaminants may pose a serious health risk. Gamma and UV irradiation are also inadequate, as these techniques may cause lipid peroxidation, which severely alters the structure of the lipid components. The remaining methods, filtration and sterile manufacturing, are time and money consuming [111].

Decades of research make liposomes a trustworthy drug delivery system. Nevertheless, non-liposomal nanocarriers are trying to overcome some of the flaws of liposomal formulations and are receiving increased attention as potential AMP delivery systems. We review some advances in the most relevant non-liposomal lipid-based NPs hereunder.

### 2.2. Lyotrophic Liquid Crystals

**Figure 2:**
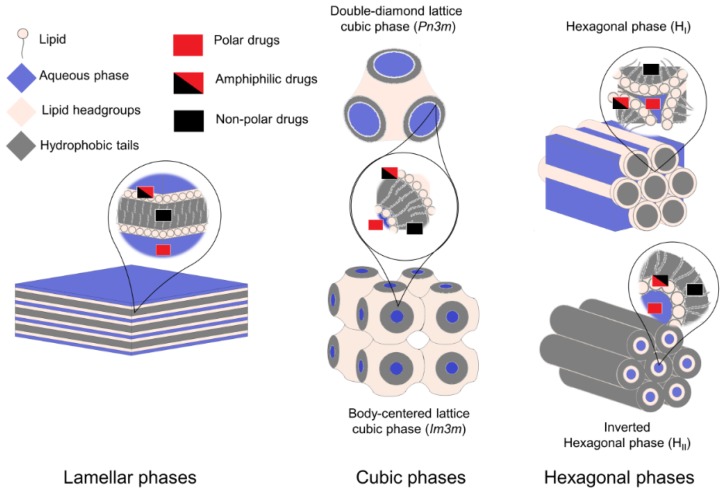
Commonly found lyotropic liquid crystalline lipid phases and preferential drug localization according to its polarity. Different phases can be achieved by varying lipid composition, water content, temperature and stabilizer additives, such as poloxamer 407. Polar or hydrophilic drugs locate preferentially in the aqueous bulk, while non-polar drugs tend to accumulate at the hydrophobic phase formed by the lipid tails. Amphiphilic molecules such as most antimicrobial peptides (AMPs) tend to locate at the interface of the polar and non-polar phases, between the lipid headgroups and the acyl chains of the lipids.

Certain lipids, such as glycerol monooleate (GMO), can form liquid crystals that can organize in several phases, such as lamellar, hexagonal and cubic phases, as illustrated in Figure 2. The adoption of one or another liquid crystalline phase depends on several environmental factors, including lipid composition, water content, temperature and additives [113]. Liquid crystals (LC) behave macroscopically as fluids, but have a highly organized crystal-like nanostructure [114]. The intricate nanoarchitecture of the liquid crystalline phases favor low diffusion coefficients of the molecules entrapped, thereby offering the potential of a sustained delivery. However, their viscosity is still too high to be suitable for parenteral administration [115]. A strategy to overcome this is to disperse the crystals with the aid of a stabilizer, such as poloxamer 407, forming liquid crystal nanoparticles (LCNPs) [115,116]. LCNPs with cubic phases and hexagonal phases are known as cubosomes and hexosomes, respectively. LCNPs are a relatively recent DDS, and their potential as AMP nanocarriers has only begun to be studied in recent years [117,118].

Boget et al. [117] studied the effects of charge and hydrophobicity of AMPs on LC architecture. The authors noticed that the most hydrophobic peptides induced an increase in the negative curvature of the cubic LC systems, while the most polar peptide induced a decrease in the negative curvature. The hexagonal phase was the most robust, but compromised the antimicrobial activity of the AMPs, while the cubosomes preserved the antimicrobial activity of the AMPs tested. Gontsarik et al. [119] showed that the addition of LL-37 to cubosomes dramatically altered their phase behavior, transforming them into vesicles and micelles. In a related study, Gontsarik et al. [120] studied the combined effect of LL-37 addition and pH alterations in the phases of LCNPs. This knowledge is crucial to be able to perform a controlled pH-triggered release of the content of the LCNPs. They found that the phase behavior strongly responds to alterations in pH in the presence of LL-37. The increase in pH resulted in alterations in the geometry of the system, due to alterations in the protonation state of the oleic acid (OA) component of the LCNPs [120].

Bernegossi et al. [121] developed a LC system to protect the antibiofilm AMP KSL-W from degradation. They found that the LC carrier was an effective platform for AMPs, displaying 100% inhibition of multispecies oral biofilm. Importantly, the system displayed suitable mucoadhesive properties in bovine teeth blocks, suggesting that strategies based on LCNPs could be useful for buccal administration of antibiofilm peptides. The major drawbacks associated to these systems are related to their short shelf life [115]. Freeze drying methods for conservation may be used; however, the conditions for the reconstitution of LCNPs must be highly controlled, as temperature changes may result in different crystal nanostructures, thus altering their properties. Furthermore, for an efficient AMP delivery, pore size control is necessary. However, to date, pore size tuning remains to be achieved [115].

### 2.3. Solid Lipid Nanoparticles and Nanostructured Lipid Carriers

**Figure 3:**
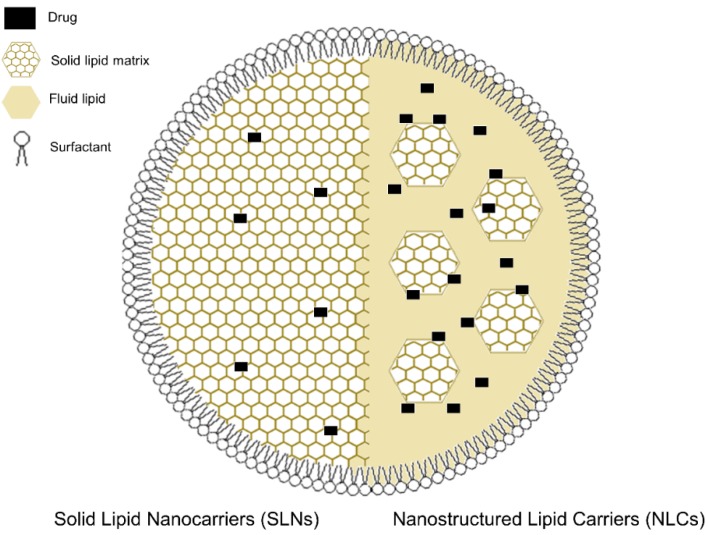
Schematic representation of the structure of a solid lipid nanoparticle (SLNs; left half) and a nanostructured lipid carrier (NLCs; right half). In SLNs, the localization of the loaded drugs is much more restricted, due to the solid lipid matrix that makes up its core, which usually translates into lower encapsulation efficiencies. The inclusion of a fluid lipid besides the solid lipid matrix in NLCs usually results in an increased drug load capacity.

Other non-liposomal LNP system that are increasingly attracting attention as potential DDS for AMPs are solid lipid nanoparticles (SLNs) and nanostructured lipid carriers (NLCs). These nanoparticles were developed more recently as alternatives to liposomes with improved stability, shelf life, encapsulation efficacy and feasibility of large-scale manufacturing. SLNs are formed by a matrix of solid lipid particles enclosed by biocompatible surfactants [122]. NLCs, on the other hand, are made up by a matrix of solid lipid immersed in oil droplets, and then also stabilized by surfactant [123]. A representation of the structure of SLNs and NLCs is presented in Figure 3. In general, the drug loading capacity of NLCs is higher, the drug diffusion in SLNs is lower and nonuniform, which has been pointed out as a disadvantage of SLNs relative to the more recent NLCs [124]. Both SLNs and NLCs are suited for oral, parenteral and ocular administration, but have been especially successful for dermal drug administration. Indeed, many marketed lipid nanoparticle-based formulations are in the cosmetic field [125].

Moreno-Sastre et al. [126] compared NLCs and SLNs as platforms for the delivery of colistin against *P. aeruginosa*. They found that nanostructured lipid carriers could have a stability of up to one year in optimized storage conditions. In a recent study, Lewies et al. [127] studied the combined effect of nisin with a series of conventional antibiotics using nanostructured lipid carriers, finding that this bacteriocin has a synergistic effect with many antibiotics, noteworthily with novobiocin. Interestingly, they also found that the inclusion of EDTA enhanced the antimicrobial activity of nisin. Sans-Serramitjana et al. [128] compared the efficacy of colistin encapsulated in SLN and in NLC. Both nanoencapsulated formulas showed similar encapsulation efficiencies and release profiles. However, the NLC-colistin formulation was more effective at killing *P. aeruginosa* and more stable through time and at different storage temperatures.

The main weakness of SLNs and NLCs is the low entrapment efficiency of polar drugs, due to the lipophilic nature of the lipid ingredients. To improve the loading capacity, different strategies have been designed, such as the double emulsion technique for SLNs [129], or the hot and cold high pressure homogenization technique for NLCs [130].

## 3. Metal Nanoparticles

Nanoparticles of noble metals have been synthesized for many applications. They are recognized by their outstanding antimicrobial properties by themselves and have been used for centuries to treat infections [131,132,133]. Due to their small size and notably high surface area-to-volume ratio, among other properties, they are attractive conjugate platforms to improve AMP efficacy [134,135]. Among all metal NPs, silver (Ag) and gold (Au) NPs are extensively investigated regarding their strong antimicrobial potential [136]. Thus, we focus on Ag and Au NPs, highlighting the recent strategies to develop peptide-NP conjugates.

In comparison with the current DDSs, metal NPs are very effective to detect molecules on a molecular scale and can offer major advantages for therapeutic and diagnostic applications [137,138]. Highly sensitive detection systems based on AuNPs and AgNPs show improved sensitivity to detect the presence or absence of a specific target [139,140,141].

**Figure 4:**
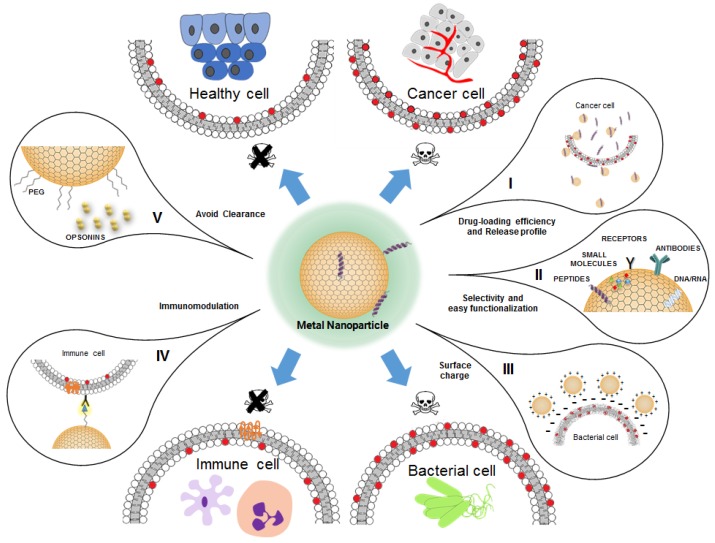
Strategies to deliver antimicrobial peptides (AMPs) using metal nanoparticles. Metal nanoparticles can be covered with a variety of chemical groups and (bio)molecules to enhance their delivery abilities. (**I**) The plasma membranes of cancer cells have anionic phospholipids exposed on the outer leaflet. The antitumor effects of MNP is determined, among other factors, by their passive targeting towards the cancer cells surface, with the help of cationic AMPs. MNP can improve the deposition and distribution of higher local doses of AMPs at the tumor site. (**II**) By active targeting, MNP can become selective to different types of cells or microorganisms. They can be functionalized with biomolecules or other ligands that would preferentially bind to cell surface receptors or other proteins, antibodies and/or DNA/RNA to gene delivery and silencing purposes. (**III**) The adsorption of MNP on bacteria membranes may lead to specific ion toxicity (membrane depolarization, perturbation of redox balance or membrane damage). Changes occur on the negatively charged cell surface, increasing its permeability. (**IV**) The ability of peptides, proteins or monoclonal antibodies to trigger the immune system, inducing a robust immune response, can be applied in the design of vaccines. (**V**) The nanoparticle surface modification with biocompatible polymers (e.g., PEG) may avoid its inactivation by the reticuloendothelial system. Furthermore, it can improve the stability, increase the solubility, decrease the cytotoxicity and enhance the bioavailability of a given drug.

Metal-based nanoparticles (MNPs) are colloidal particles with exclusive properties. They possess specific optical behavior, electrical conductivity, and high thermal and chemical stability, which bulk forms do not possess [134,142]. A major advantage of MNPs derives from their ability to be modified into bioconjugates [143,144]. By using different molecules, it is possible to alter MNPs so that they can fulfil the criteria as efficient drug delivery agents (Figure 4), which implicates high stability between the drug and the NP, low toxicity and immunogenicity, affinity to the target, controlled release, safe degradation and, ultimately, reduced health-care costs [145]. MNPs are generally unstable in suspension. It is important to prevent NPs from binding between each other in order to avoid aggregation phenomena [146]. The problem associated with this is compromising the results due to the instability of NPs in culture media. These media contain salts that may alter the size and charge of a particle and affect ion release, leading to alterations in the NP biodistribution and irreproducible results [146]. An important aspect of MNPs is their bioconjugation by using a variety of surface functionalization molecules, which may help the peptide-NP conjugate to cross the multiple biological barriers they face and protect them from mechanisms of immune recognition and cellular clearance [135]. Based in the principle of multivalence, the unique interactions between peptide and NP surface provide an improved selectivity [147]. Depending on the NP shape, size and surface chemistry, the binding of a functionalized nanoparticle can promote or enhance specificity to target cells or cell surface molecules [37]. Selective delivery scaffolds of AMPs conjugated with AgNPs or AuNPs are a promising and prolific field of investigation. Recent developments in AMPs-AgNP/AuNP conjugates show the extended nanoparticle morphology that has been tested against a wide variety of microorganisms (summarized in Table A1). Their particular characteristics make them suitable for chemotherapy, as they can be internalized specifically by cancer cells, avoiding toxicity for healthy cells [148].

Particle size and size distribution have a major influence in the internalization of NPs, as well as in their functionalities, directly affecting NP toxicity and particle distribution in vivo [149]. Smaller metal-based NPs have a higher surface area-to-volume ratio and are more effective, due to increased contact surface, reducing toxicity. The smaller diameter is related to enhanced microbicidal activity [149]. Due to the clearance by phagocytes of particles above 200 nm, smaller nanoparticles usually have a higher lifetime in circulation, without being recognized by immune cells. Moreover, they can leave the tissues easily by extravasation or renal clearance, avoiding accumulation in the liver, spleen and other organs [39].

MNPs can take several forms. The most common is the spherical, but one can find some studies using cubes, stars, rods, cones and cages [150,151]. However, the principal factor in NP shape is the surface-to-volume-ratio. This property reflects the surface area available for the biomolecules to bind. Functionalization with an abundant functional ligand enables multivalence on the MNP surface, ensuring the conjugate binding to the target [134]. MNPs always present some associated polydispersity that can affect their biological activity. Niikura et al. [152] prepared spherical, rod and cubic AuNPs with different sizes and coated them with West Nile virus envelope protein (E). They evaluated whether AuNP-Es could act as vaccine adjuvants. They showed that AuNP-Es have size/shape-dependent mechanisms and that they can modulate the immune response to produce cytokines and antibody in different ways among the AuNP-Es shapes tested, reveling the influence of the surface area on the specific MNPs activity and cytotoxicity.

Concerning the charge of MNPs, positively charged NPs usually display improved cell uptake by electrostatic interaction with negatively charged cell membranes [153,154]. Due to AMPs cationic and amphipathic sequences, they are easily attracted by the negatively charged components found in the outer leaflet of the bacteria membranes, making them highly selective to external pathogens [155,156]. In the case of AMP-AgNP/AuNP conjugates, the disadvantage is the negatively charged nucleus of eukaryotic cells and the cellular toxicity associated to healthy cells [154]. MNPs can selectively target membranes with negatively charged glycocalyx alterations, like in some cancer cells; for this application, slightly positively charged MNPs are more indicated to specifically target the tumor site [153].

MNPs can be internalized into cells by different pathways, with the mode of entry depending on their physicochemical properties and the specific microenvironment targeted by the MNP [157]. The mode of entry into the cell is important for NPs design to target intracellular molecules or pathogens. Furthermore, NPs need to overcome the natural barriers of the host before entering the bloodstream, to be later on internalized by the target cell [157]. Xie et al. [158] investigated the celular uptake of AuNPs coated with methylpolyethylene glycol (mPEG), with three different shapes, by RAW264.7 cells. Using endocytic inhibitors, they were able to identify distinct endocytic pathways to internalize each AuNP morphology [158]. Due to their nanoscale size, metal NPs can enter mammalian cells and can also cross the blood-brain barrier [27].

Lower MICs, high stability, lower toxicity to host cells, absence of deposition in tissues, non-hemolytic and non-immunogenic are requirements to develop an efficient AMP-NP conjugate. To achieve this, the control over the structural parameters during the synthesis is crucial. Inorganic materials as silver and gold nanoparticles have relatively simple synthesis methods. There are various types of synthesis, but the most used methods are chemical, physical and biological. The concentration of metal salt, reducing agents, pH, temperature and time play important roles in the synthesis, in order to obtain the desired physicochemical properties for a given application [136]. The formation of nanostructures can be made by the so-called “top down” and “bottom up” methods. The top down approach uses macroscopic structures that are reduced to the nanoscale [159]. The bottom up approach, in contrast, starts with atoms or molecules, associated afterwards in order to reach the nanoscale. This method allows more control over the “seed” (e.g., primarily formed nanoclusters) growing, is considerably less expensive and results in a colloidal suspension [160,161]. The most widely used method for spherical AuNPs production was developed by Turkevich et al. [162] in 1951. It is based on the reduction of HAuCl_4_ (tetrachloroauric acid) by sodium citrate in water. This method results in small AuNPs of about 20 nm in diameter. In this technique, citrate ions play a double role, both as stabilizing and reducing agents. The Turkevich method was slightly modified by Frens et al. [163] in 1973, and has been further modified by several research groups, becoming the most commonly used method for AuNPs synthesis. For AuNPs, the most common shape is spherical, but other shapes, such as stars, plates, tubes, cubes, rods and triangles, have also been reported [144,158]. The most common approach for synthesis of silver NPs is single-phase chemical reduction. Although silver and gold have distinct characteristics, the synthesis methods are, in general, similar. Using different reducing agents for the reduction of silver ions (Ag^+^) in aqueous or non-aqueous solutions, metallic silver (Ag^0^) is formed, followed by the seeds’ formation [161]. The use of stabilizing agents protects the suspension from aggregation. Surfactants like thiols, amines, acids and alcohols are usually used for this purpose [164].

The principle of the chemical reduction is based on the use of a strong reducing agent, such as sodium citrate, tannic acid, ascorbate or sodium borohydride [165,166]. In solution, these chemicals act by reducing the metal salt to form a seed of stable metal nuclei. The selection of a reducing agent is determinant, as the diameter and the size distribution of the MNPs will depend on the reducing agent used. The bottom up method can generates reproducible results, enabling the preparation of colloidal NPs with a controlled shape and narrow size distribution [161]. However, the most common method of production requires the use of products highly harmful to the environment [167].

The well-known physical methods for metal NPs production are microwave, evaporation-condensation and laser ablation [167]. The advantages are large-scale faster production, the production of smaller NPs and the absence of solvent contamination. However, synthesis of MNPs requires high-energy consumption, more time to reach thermal stability and more space for production. Biological or‘green’ methods employ microorganisms such as prokaryotic bacteria and eukaryotic fungi or living plant extractsas reducing agents [168]. They all have constitutive bioactive polyphenols, alkaloids, proteins, sugars and phenolic acids, among other, and can act both as stabilizing and reducing agents. This approach can provide longer stability to the NPs by mimicking the nature method and has been pointed out as a more ‘eco-friendly’ method that avoids releasing high amounts of toxic substances into the environment. On the other hand, its disadvantages include the wide distribution in particle size and the purification, as there may be contamination with bacteria or other cellular components [160].

As for the biocompatibility of MNPs and their immunoreactivity, the problematic resides in the misleading results from the NP immunomodulatory effect. As soon as NPs enter into circulation, they rapidly meet the mononuclear phagocytic system in special components of the innate immunity, such as antigen-presenting cells [169]. MNPs may activate the innate immune response via Toll-like receptors and activate macrophage inflammasome-dependent cytokine secretion to produce interleukin-1β (IL-1β) and IL-18 [170,171]. AgNP and AuNP can activate the cellular and humoral immune response, inducing the production of both pro-inflammatory (IL-1, IL-6 and tumor necrosis factor α (TNF-α)) [172,173] and immunosuppressor cytokines (IL-10 and transforming growth factor β (TGF-β)) [174,175]. Thus, they can be used for immunization therapies [176]. Staroverov et al. [176] investigated the immunization of animals mediated by antigen–AuNP conjugates for swine transmissible gastroenteritis virus (TGEV). The production of cytokines evaluated was higher in TGEV antigen-AuNPs conjugates-treated animals, when compared with control or animals injected with the antigen alone. The presence of IL-1β can be correlated with macrophage activity and stimulated B cells, and higher concentrations of IL-6 are stimulatory to cellular immunity in animals immunized with TGEV antigen-AuNPs [176]. On the other side of immunotherapy, the use of metal-based nanoparticles for immune response evasion has been explored to treat autoimmune diseases. Dul et al. [177] conjugated an autoantigen, the PIC19-A3 peptide, with AuNPs for the treatment of type 1 diabetes. They showed that the uptake of the peptide-AuNP conjugates by dendritic cells (DCs) prevents subsequent T-cell priming and activation. This effect is due to DCs remaining in the immature state adopting a suppressive effect rather than an inflammatory phenotype. More studies are necessary to understand how NPs interact with the immune system and how this interaction could affect DCs, not only focusing in NP characterization, but also addressing other relevant issues, such as immunocitotoxicity.

### 3.1. Antimicrobial Peptide (AMP)-Conjugated Metal Nanoparticles against Bacteria Infections

It is believed that the use of metal nanoparticles is important to avoid the development of bacteria resistant strains, since bacteria fail to mount a defense against the mechanisms of action of MNPs [151]. Despite many efforts, the specific pathways responsible for this activity are poorly understood, but they have been related to the structural and morphological changes in bacteria cells [132]. When MNPs interact with bacteria, they can be attached to the cell surface, compromising the integrity of the cell wall and entering the cytoplasm [164]. Through the release of ions in the intracellular environment, MNPs disrupt the respiratory chain machinery, further increasing bacteria cell permeability. MNPs can also stimulate oxidative stress and ROS production, inhibiting ATP production and bacteria DNA replication, finally leading to cell death [82,132,152].

Silver is an attractive material, well known for its natural activity against both Gram-negative and Gram-positive bacteria, acting in multiple pathways [132]. It is the most popular noble metal used in nanoparticle synthesis. It has distinctive properties, such as surface-enhanced Raman scattering, optical behavior, good electrical conductivity, small size and high surface area-to-volume ratio. Those characteristics results in increased reactivity, chemical stability and antimicrobial activity, allowing its use in many applications [178]. AgNP can be specifically conjugated with antimicrobial peptides to treat bacterial infections. Although these are good possibilities for infection fight, only five AgNP formulations combined with anti-infective drugs are currently under clinical trials [179].

Liu et al. [165] recently described a short amphiphilic cell penetrating peptide (G3R6TAT) as the stabilizer and reductant to produce AgNPs. They have reported an enhanced antimicrobial effect towards bacteria (the Gram-positive *Bacillus subtilis* and Gram-negative *Escherichia coli*) and fungal pathogens (*Candida albicans*), with low hemolytic activity at effective concentrations of the peptide-AgNP conjugate, when compared with the AgNP alone. However, the mechanism of the interaction between the peptide and the NP was not characterized. In 2009, Ruden et al. [180] used polymyxin B, effective against Gram-negative bacteria, combined with AgNPs. Polymyxin B acts synergistically with AgNPs when tested against several Gram-negative bacteria. In fact, the PB-AgNO_3_ combination induces hemolysis, but, when conjugated with AgNPs, it does not display hemolytic activity, even at high concentrations. With a similar approach, Mei et al. [181] synthetized spherical AgNPs functionalized with the AMPs bacitracin A and polymyxin E (AgNPs-BA&PE) against *E. coli*, *P. aeruginosa*, *S. aureus* and *Bacillus amyloliquefaciens.* They further investigated the antibacterial mechanism of AgNPs-BA&PE, revealing that the conjugate enters the bacteria by binding to Ca^2+^ and Mg^2+^ in the outer membrane. Once inside, they disrupt the membrane, leading to cytoplasm leakage. *P. aeruginosa* and *S. aureus* bacteria resistance was also tested. The MIC remained constant and AgNPs-BA&PE did not induced resistance. Another study, by Mohanty et al. [182], used the AMPs NK-2 and a LL-37 variant named LLKKK-18, conjugated with two biogenic AgNPs (NP-1 and NP-2) against mycobacteria (*Mycobacterium smegmatis* and *M. marinum*). The combination with labeled AgNPs shows the internalization of both AgNPs by mycobacteria. The results demonstrated similarly increased intracellular killing of *M. smegmatis* for NK-2 combination with both NP-1 and NP-2, when compared with the molecules alone. However, LLKKK-18 showed antibacterial activity only with NP-2. This study reveals size-dependent outcomes and another possible killing pathway for AgNPs in a nitric oxide-independent manner.

The synthesis of the AgNPs in the presence of the peptide could be used for their functionalization, as well as a stabilizer agent for the NPs assembly. The AgNP surface chemistry plays an important role in the development of a successful AMP-NP conjugate [178]. To confer stability to these systems, cysteines (Cys) have been used to stabilize the interactions of proteins with AgNPs [183]. Pal et al. [183] showed the importance of surface stability by conjugating the peptide odorranain-A-OA1 (OA1), an AMP from the skin of the Chinese odorous frog, containing two Cys residues, with 10 nm AgNP (AgNP-OA1). The antibacterial activities of the AgNP-OA1 conjugates were evaluated on Gram-negative *E. coli* cells and using vesicles mimicking Gram-positive bacteria membranes. Using different techniques to assess the antimicrobial activity of the conjugate, the results showed that AgNP-OA1 enhanced bacteria leakage as compared to the free peptide. Regarding biocompatibility, the conjugate did not show any significant cytotoxicity. Taken together, AMPs-AgNP conjugates act synergistically to enhance antibacterial activity via different pathways without affecting mammalian cells. Continuing the investigation in AMPs-AgNPs conjugates, Pal et al. [184] recently published a study describing a potent AMP, andersonin-Y1 (AY1), and its AgNP conjugate, against multidrug resistant strains *Klebsiella pneumonia*, *P. aeruginosa* and *Salmonella typhi*. In this study, they modified AY1 by adding Cys either at the N-(CAY1-AgNP conjugate) or at the C-terminal (AY1C-AgNP). Both combinations resulted in increased stability and antibacterial activity against the multidrug-resistant strains.

Gold nanoparticles have also been used in a wide variety of applications. Nevertheless, their potential use associated with AMPs against bacteria has proven to be an interesting strategy to overcome AMR [185,186,187]. Sharing all the structural properties with AgNPs, AuNPs are also versatile and can be produced in different sizes and shapes. The optical characteristics of AuNPs, namely localized surface plasmon resonance (LSPR), in which AuNPs absorbs and emits in the near-infrared (NIR; 650–900 nm) is specially used in diagnostics, in vivo imaging of target cells and radiotherapy [145,188]. To further understand the antimicrobial effect of AuNPs, Lee et al. [189] used an AMP, HPA3P^His^, loaded onto a gold nanoparticle-DNA aptamer (AuNP-Apt) conjugate (AuNP-Apt-HPA3P^His^) to show in vitro and in vivo the effectiveness of peptide intracellular delivery. They demonstrated that AuNP-Apt-HPA3P^His^ conjugates improve the penetrability of HPA3P^His^ and eliminates bacteria a few hours after treatment, without affecting the host, in comparison with the peptide alone. The bactericidal action of AuNP-Apt-HPA3P^His^ was improved and used as a therapeutic construct against *Vibrio vulnificus* infection. Since the mortality rate is over 50% among infected patients with sepsis, the interaction with immune cells was not discussed in this study, and the lack of a systemic infection model to evaluate a late stage of the infection would be a great addition to consolidate the conjugate as a drug delivery system. Another in vivo study by Rai et al. [190] proposed a new AMP-conjugated NP with the peptide cecropin-melittin (CM-SH-AuNPs). They tested the conjugate against *S. aureus*, *E. coli*, *P. aeruginosa* and *K. pneumonia*, and demonstrated that the conjugate is more efficient than the free peptide in inducing the permeabilization of bacteria cell membranes. The in vivo action of CM-SH-AuNPs was tested in animal models to demonstrate decreased bacteremia and a low systemic inflammatory response, proving to be non-immunogenic, non-hemolytic and non-cytotoxic to human cells.

Casciaro et al. [191] recently investigated PEGylated gold nanoparticles (AuNPs@PEG) functionalized with Esc(1–21) (AuNPs@Esc(1–21)), against the motile and sessile forms of *P. aeruginosa*. AuNPs@Esc(1–21) are responsible for disrupting the bacteria membrane and killing likewise Esc(1–21) alone. AuNPs@Esc(1–21) present antibiofilm activity, with about 50% killing of biofilm cells. An important finding was the maintenance of antimicrobial activity even in the presence of the proteolytic enzyme trypsin and, for the first time, the wound healing activity of the peptide conjugated AuNPs was demonstrated. Besides their antimicrobial activity, AuNPs are seldom used for diagnostics applications. Miranda et al. [192] developed an electrochemical biosensor for detection of Gram-negative bacteria. For this, they used the peptide clavanin A (ClavA) with Cys-modifed AuNPs (AuNPsCys) and the best results were obtained from *Salmonella* Typhimurium and *E. coli* strains. They analyzed the reaction between ClavA and bacteria and concluded that the biosensor has sensibility and specificity to differentiate Gram-negative bacteria.

Once the community of a biofilm is formed, it is far from the antibiotic and immune system mediators reach. For the treatment of these pathogens, high doses of antimicrobial drugs are needed, due to resistance of the microorganism, eventually leading to undesirable side effects. Metal NPs have antibiofilm activity, as they can penetrate into bacteria and interact with the biomolecules and cellular structures, finally leading to bacteria membrane disruption and death [193]. Alteriis et al. [194] described how AuNPs coated with the AMP indolicidin were able to penetrate into the *C. albicans* biofilms matrix and inhibit their early formation, eradicating mature biofilms in cell lines and in clinical isolates from medical devices and blood. AgNPs have the potential to optimize orthopedic implants, which are fundamental to the treatment of diverse lesions [193,195].

### 3.2. Anticancer Antimicrobial Peptide (AMP)-Conjugated Metal Nanoparticles

Based on electrostatic bonding, cationic AMP-MNPs can selectively bind to the bacteria surface and specifically recognize transformed cancer cells [196,197,198]. These cells lose their asymmetric transmembrane distribution of lipids and display a negatively charged surface, due to the increased proportions of phospholipids with negatively-charged headgroups, such as phosphatidylserine in the outer leaflet of the plasma membrane [196]. MNPs can inhibit cell growth and mediate cell death by mechanisms not completely understood up to date. Together, the conjugate can access the tumor microenvironment and remain unrecognised by the immune cells.

Peptides conjugated with silver or gold nanoparticles enhance their stability when interacting with colon and breast cancer cells, decreasing the death rate through a controlled delivery of the anticancer peptides [199]. AgNPs anticancer properties have been tested in vitro in different cell lines, demonstrating potential cytotoxic, antiproliferative and apoptotic properties against some cancer cells [200]. As previously indicated, AuNPs have unique optical and thermal properties. Although their research field is well established, there are few examples of AuNPs being actively investigated in clinical trials and there is none approved by the FDA until now. This is likely due to the affinity of gold to DNA and impairment of normal cell function. To overcome this problem, the combination of nanoparticles with other substances can be relevant [21]. In chemotherapy, AuNPs have been mainly used associated with cell penetrating peptides [201]. There are many studies exploring the anticancer properties of the AuNPs as DDSs for selective target of biomarkers, as probe for contrast agent and as thermal agent for PDT [131,140,145,202]. Difficulties remain in the NP size/shape dispersity, relevant dose delivered to the tumor and the low number of studies exploring AMPs-MNPs formulations in cancer; together, these factors are the major challenge for the success of new DDSs based on metal scaffolds [145,203].

The synergistic effect of AMP-conjugated metal nanoparticles is achieved in part by the therapeutic properties of the NPs themselves, and not only by their fulfillment of the requirements as good carriers [132,137,145,146]. The wide variety of forms and size distributions has been proven to be an advantage of MNPs; however, the challenge remains in developing a standard MNP [146,161]. Keeping the same synthesis conditions for each batch to maintain the structural characteristics from the synthesis method used is fundamental. This double-edged sword is amplified by size-related cytotoxicity, immunoreactive residues from synthesis, lack of methods to predict the immunocompatibility, insufficiency of studies on immunotoxicity, reproducibility, challenges related to the physicochemical characterization and, finally, translation from mice to human patients [39,49].

## 4. Conclusions and Future Perspectives

Worrisomely, bacteria evolve faster than we develop new antibiotics [204]. We have a potentially unexploited arsenal of antibiotics in AMPs. Nonetheless, the poor pharmacokinetics of peptide drugs is limiting their use. The combination of AMPs with drug delivery systems such as liposomes and metal nanoparticles can be fundamental for their implementation. At this point, the development of suitable nanoparticles to deliver AMPs is a major bottleneck for the implementation of AMPs in the clinical practice [137]. Indeed, the majority of nanodrugs under clinical trials are anticancer and antimicrobial, revealing the increasing interest in this matter [205].

Since 1995, up to 50 nanopharmaceuticals have received FDA approval. The search of the term ‘nano’ yields 95 active clinical trials in the Clinical Trials website (May 2019) [206]. A large tendency is for micelles and protein-based NPs, while an increased use of metal NPs can also be noticed [207]. Peptides are still one of the major pharmaceutical market targets, but their future as drugs is still fragile. Thus, the upcoming Magic Bullet might well be a nanoparticle conjugated with AMPs.

Despite the great advances in nanomedicine and its rapid growth, there are still several obstacles to their progression to clinics. A large gap between the encouraging in vitro results, the rather disappointing pre-clinical results, and the low impact in clinical settings can often be found. NP-host interactions are difficult to tackle. In pre-clinical studies, it has been pointed out that the need of immunodeficient mice for xenograft tumor transplantation may be leading us to overlook immune interactions that hamper NPs activity. Sound strategies are needed to investigate cytotoxicity, inflammatory response and immune response, not only in cell culture, but also in clinical isolates, as there is a need for further in vivo studies to ensure the safe use of NPs. Long-term toxicity studies are also needed to better understand the implications of silver and gold deposition in the human body. The use of lipids in nanoparticles offers more possibilities of drug encapsulation and increases the possibilities of acceptance as DDSs by the regulatory agencies, due to their biodegradability and common occurrence in biological systems.

Based on the findings reviewed here, research and clinics should come together to improve the potentiality of these DDSs, which may have an important role in human healthcare. It is imperative to overview the approaches of AMP-NP formulations to improve therapeutics and reduce side effects.

## Supplementary Materials

The following are available online at https://www.mdpi.com/1999-4923/11/11/588/s1, Table S1: Clinical trials of AMPs and proposed applications.

## Abbreviations

Ag	Silver
AMP	Antimicrobial peptide
AMR	Antimicrobial resistance
ATP	Adenosine triphosphate
Au	Gold
AY1	Andersonin-Y1
Chol	Cholesterol
CL	Cardiolipin
Clav A	Clavanin A
CS	Chitosan
Cys	Cysteine
DC	Dendritic cell
DDS	Drug delivery system
DLS	Dynamic light scattering
DNA	Deoxyribonucleic acid
DPPC	Dipalmitoylphosphatidylcholine
Epi	Epirubicin
EPR	Enhanced permeability and retention
FDA	USA Food and Drug Administration
GDNF	Glial-derived growth neurotrophic factor
GMO	Glycerol monooleate
HDP	Host defense peptide
IL	Interleukin
LC	Liquid crystal
LCNP	Liquid crystal nanoparticle
LNP	Lipid nanoparticle
LPS	Lipopolysaccharide
MIC	Minimum inhibitory concentration
mPEG	Methylpolyethylene glycol
MNPs	Metal-based nanoparticle
MRSA	Multi-resistant *Staphylococcus aureus*
NLC	Nanostructured lipid carrier
NP	Nanoparticle
OA1	Odorranain-A-OA1
OA	Oleic acid
PB	Polymyxin B
PC	Phosphatidylcholine
PDT	Photodynamic therapy
PE	Phosphatidylethanolamine
PEG	Polyethylene glycol
PG	Phosphatidylglycerol
POPC	1-palmitoyl-2-oleoylphosphatidylcholine
PVA	Polyvinyl alcohol
PVP	Polyvinylpyrrolidone
ROS	Reactive oxygen specie
SLN	Solid lipid nanoparticle
TGEV	Transmissible gastroenteritis virus
TGF-β	Transforming growth factor β
TNF-α	Tumor necrosis factor α
UHT	Ultra-high temperature processed

## Appendix A

**Table A1:** AMP-metal nanoparticle conjugates studies and applications.

AMPs	NPs	Shape	Diameter (nm)	Applications	Reference
Polymyxin B Gramicidin S	AgNPs	-	25	Synergic activity against *E. coli*, *Acinetobacter calcoaceticus*, *Enterobacter helveticus*, *Aeromonas bestiarum*, *Proteus myxofaciens*, *Pseudomonas fluorescens*, *Bacillus subtilis*, *Kocuria rhizophila* and *Micrococcus luteus* Synergic activity against *E. helveticus*, *P. myxofaciens* and *P. fluorescens*	[180]
Polymyxin B	AuNPs	Spherical	2.7 ± 0.7	Maintains the same antimicrobial activity as the free form of polymyxin B against *E. coli* and methicillin-resistant *S. aureus* (MRSA)	[208]
Nisin	AuNPs	Spherical	12.0 ± 2.0	*M. luteus*	[209]
NK-2 LLKKK-18	AgNPs-*Alstonia macrophylla* biomass AgNPs-*Trichoderma* sp. Biomass	Spherical	50 and 100	*M. smegmatis* *M. smegmatis* and *M. marinum*	[182]
Bacitracin A and polymyxin E	AgNPs	Spherical	3.1	*E. coli*, *B. amyloliquefaciens*, *P. aeruginosa* and *S. aureus* Promotes healing of infected wounds	[181]
G_3_R_6_TAT	AgNPs-citrate AgNPs-SDS and Au@Ag-BSA	Triangular Spherical	30–70 30	*B. subtilis*, *E. coli* and *C. albicans*	[165]
LL-37 CYS-modified (LL37-SH)	AgNPs	Spherical	5.3 ± 1.8	No anti-proliferative effect on primary skin cells; promotes wound healing, preventing potential infection by *E. coli*, *Staphylococcus epidermidis*, *S. aureus*, and free living and biofilm forms of *P. aeruginosa*	[210]
Nisin	AgNPs incorporated in poly(d,l-lactide) (PDLLA) and poly(ethylene oxide) (PEO) nanofibers	-	21.81 ± 5.5	*P. aeruginosa*, *Klebsiella pneumoniae*, *E. coli*, *S. typhimurium* and *S. aureus*	[211]
RPT-0001	AgNPs	Spherical	20–30	Against food-borne bacterial pathogens: *L. monocytogenes*, *Cronobacter sakazakii*, *S. enterica* subsp. *enterica* and *E. coli*	[212]
Indolicidin	COOH-functionalized AuNPs	Spherical	3	Immuno suppressive action by downregulation of IFNβ expression and increase of IL-10 in RAW264.7 murine macrophage cells and THP-1 human monocyte cell lines	[213]
OA1	AgNPs-citrate	Spherical	10	*E. coli*	[183]
PEP (a peptide sequence from lactoferrin)	AuNPs-polyethylenimine (PEI)	Spherical	-	Carrier for in vivo gene delivery vector in MSCs cells. Antibacterial activity against*S. Aureus*, both in vitro and in vivo.	[214]
Nisin	AgNPs	Spherical	10.1 ± 1.7	*B. subtilis*, *E. coli*, *Proteus vulgaris* and *S. aureus*	[215]
LL37-SH	AgNPs with type I collagen as capping agent	Spherical	4	Sprayed formulation against free living and biofilm forms of *P. aeruginosa*	[216]
Hexahistidine-tagged A3-APO (A3-APO^His^)	AuNPs-DNA aptamer	Spherical	15	Deliver of AMPs to *Salmonella enterica* and Typhimurium-infected HeLa cells	[217]
α-lipoic acid-peptide (LA-WKRAKLAK)	CTAB^I^-capped AuNPs	Spherical Rod	28.1 and 49.7 20 and 40	Resistant cancer cells MCF-7 and metastatic T47D breast cancer cell line	[218]
Cecropin-mellitin	AuNP-coated SPIONs^II^	Quasi-spherical	12 ± 2 (gold layer: 3)	*E. coli* and *S. aureus*	[219]
Cecropin-melittin (CM) CM-SH(cysteine at C-terminus)	AuNPs	-	14	*S. aureus*, *E. coli*, *P. aeruginosa* and *K. pneumoniae*	[190]
Cecropin-melittin	AuNPs-cysteamine AuNPs-PEG-NH_2_	spherical	20	Coating based on CM peptide on AuNPs immobilized glass surfaces against *S. aureus* and *E. coli*	[220]
Cecropin-melitti CM-SH (cysteine at C-terminus)	AuNPs	-	-	Adsorption process of CM peptides onto a gold surface based on all-atom molecular dynamics simulations	[221]
CYRGRKKRRQRRR containing domain of trans-activator of transcription (TAT) (ANS^III^-TAT)	AuNPs	Spherical	3.8 ± 0.7 and 22.1 ± 3.6	Cancer cells HepG2, MCF-7 and resistant cancer cell line MCF-7/ADR	[222]
Esculentin-1a(1–21)NH_2_	AuNPs@PEG^IV^	Spherical	14	Free living and biofilm forms of *P. aeruginosa*	[191]
Clavanin A	AuNPs-Cys	Spherical	10	Sensitive biosensor for Gram-negative bacteria detection: *S. aureus*, *E. faecalis*, *P. aeruginosa*, *S.*Typhimurium and *E. coli* (higher levels of response were observed for the last two)	[192]
Ubiquicidin 29–41	AgNPs	Spherical	12.3 ± 3.9	*E. coli* and *P. aeruginosa*	[223]
l-Arg-l-Arg-OMe l-His-l-Arg-OMe l-His-l-His-OMe	AgNPs AuNPs	Spherical	12 ± 2 14 ± 2	AgNPs have additive effect and enhance the antimicrobial activity of the peptides, whereas AuNPs reduce their activity against *E. coli*, *S. aureus* and *S.* Typhimurium	[142]
LL37	AuNPs	Spherical	15–25	Enhances the migratory properties of keratinocytes in vitro and has higher wound healing activity in vivo (skin wound healing)	[224]
Polymyxin B	AgNPs	Spherical	2	Inhibited the growth of polymyxin B-resistant *P. aeruginosa* isolates from patients with acute exacerbations of cystic fibrosis	[225]
x-PGLa x-MSI103 x-MAP x-BP100 x-TP10	AuNPs	Spherical	5–7	The peptides change to α-helical conformation onto the NPs surface in the presence of model membranes and maintain the same antimicrobial activity as in the free form against *E. coli*, *B. subtilis*, *S. aureus* and *M. luteus*	[226]
Lycosin-I	AuNPs	Spherical Rods	60.88 ± 0.48 65.80 ± 3.18	Efficient selectivity and cellular internalization for cancer cells in vitro, and efficient accumulation in tumors in vivo Can translocate specifically into cancer cells and kill by photothermal effect under near infrared (NIR; 808 nm) irradiation in vitro and in vivo	[227]
HPA3P^His^	AuNPs-DNA aptamer	Spherical	15	*Vibrio vulnificus*	[189]
VG16KRKP	AuNPs	Spherical	20	Potent in vitro and in vivo anti-*Salmonella typhi* activity. The conjugate can penetrate into host epithelial and macrophage cells, and lysis the internalized pathogen.	[228]
LL37 Cys-modified (LL37-SH)	AuNPs	-	-	Computational study on the interaction of the AMP with a AuNP, showing that the cysteine may have an effect on the formation of the conjugate	[229]
Human β-defensin 3 (hBD3)	AuNPs	Spherical	45	Promotes the osteogenic differentiation of human periodontal ligament cells	[230]
Nisin	AgNP (green synthesis)	Spherical	233	Induce inflammatory response via increasing IL-12 without changes on the production of TNF-α by macrophage cells	[231]
Indolicidin	AuNPs	Spherical	5	Biofilm formation of *C. albicans* and *Candida tropicalis* multi-resistant clinical isolates	[194]
LL37	AuNPs with poly(ethylene imine) as capping agent	Spherical	7	Bactericidal effect in vitro with MRSA from human isolates from ulcers in diabetic patients and in vivo with diabetic wound healing models. Combined with pro-angiogenic (VEGF) plasmids, the conjugate prevented MRSA infection in wound sites.	[232]
1018-derivative peptide (1018K6)	AuNPs	Spherical	8 ± 2	Bacterial killing ability against *L. monocytogenes* (food-isolated) and *Salmonella typhi*	[233]
Andersonin-Y1 (AY1) CAY1 (cysteine at C-terminus) AY1C (cysteine at N-terminus)	AgNPs	Spherical	10	Better MIC^V^ with cysteine tagged nanoconjugates against *E. coli* and multidrug resistant strains of *P. aeruginosa*, *Salmonella typhi* and *K. pneumoniae*	[184]
Daptomycin	AuNPs	Spherical	6	Causes bacterial genomic DNA fragmentation in MRSA	[234]
Motif (Pep-H) of human neutrophil peptide-1	AuNPs	Spherical	20	Antimicrobial activity against intracellular *M. tuberculosis* in infected monocyte-derived macrophages	[235]
