# Supplementary material for: Advances in Lipid and Metal Nanoparticles for Antimicrobial Peptide Delivery
Source: Pharmaceutics. 2019 Nov 8;11(11):588. doi: 10.3390/pharmaceutics11110588 (PMC6920925; doi:10.3390/pharmaceutics11110588)
Supplement: Supplementary file 1 [file pharmaceutics-11-00588-s001.pdf]

pharmaceutics

MDPI

Supplementary Materials: Advances in Lipid and Metal Nanoparticles for Antimicrobial Peptide Delivery

Marcin Makowski, Ítala C. Silva, Constança Pais do Amaral, Sónia Gonçalves, Nuno C. Santos

Table S1. Clinical trials of AMPs and proposed applications.

AMP	Application	Clinical trial			
		Started	Ended	Ongoing	Phase
**AB103**	Efficacy and safety of AB103 in necrotizing soft tissue infections treatment	December 2015	November 2019	X	Phase 3
**AB103**	Safety, pharmacokinetics and immunomodulatory effects of AB103 in necrotizing soft tissue infection patients	December 2011	December 2012		Phase 2
**C16G2**	Safety, microbiology study and tolerability of C16G2 varnish and strip	July 2017	January 2018		Phase 2
**C16G2**	Safety and microbiology study of C16G2 in multiple oral gel and/or mouth rinse doses	February 2014	August 2014		Phase 2
**C16G2**	Safety, microbiology study and pharmacokinetics of single or multiple oral C16G2 gel doses	October 2014	July 2015		Phase 2
**C16G2**	Evaluation of the safety and microbiology of C16G2	July 2015	March 2016		Phase 2
**C16G2**	Evaluation of the microbiology and safety of C16G2 administration	November 2015	September 2016		Phase 2
**C16G2**	Evaluation of the safety and microbiology of C16G2 varnish administered in multiple doses to dental subjects	January 2017	March 2017		Phase 2
**C16G2**	Evaluation of the microbiology, safety and tolerability of C16G2 dental strip application	February	April 2017		Phase 2

		2017		
**Colistin**	Colistin-rifampin combination and colistin monotherapy in extensively drug-resistant *Acinetobacter baumannii*	October 2016	June 2018	Not applicable
**Colistin**	Safety and efficacy study of high dose colistin	May 2014	October 2016	Phase 4
**Colistin**	Combined dry powder tobramycin and nebulized colistin inhalation in cystic fibrosis patients	March 2014	November 2016	Phase 3
**Colistin**	Aerosolized + intravenous *vs.* intravenous colistin for ventilator-associated pneumonia due to pandrugs-resistant *A. baumannii* in neonates	September 2016	October 2017	Phase 3
**Colistin**	Efficacy and mortality of a loading dose of colistin in critically ill patients	April 2014	December 2018	Phase 4
**Colistin**	Optimization dose study on pharmacokinetics and pharmacodynamics of colistin in critically ill patients	October 2011	October 2013	Phase 1
**Colistin**	Colistin pharmacokinetics in continuous renal replacement therapy	November 2013	August 2015	Phase 4
**Colistin**	Therapy of ventilator-associated tracheobronchitis caused by Gram-negative bacteria with nebulized colistin	December 2015	December 2017	Phase 3
**Colistin**	Detection of colistin-resistant isolates from patients of intensive care units and oncohaematology wards	July 2019	July 2021	
**Colistin**	Evaluation of the nephro/neurotoxicity in the anti-infectious therapy with inhalative colistin therapy for patients with ventilator-associated pneumonia	September 2013	December 2015	
**Colistin**	Efficacy of ascorbic acid for prevention of colistin-associated nephrotoxicity	January 2012	December 2013	Phase 3
**Colistin**	Trial of colistin *vs.* meropenem in ventilator-associated pneumonia	May 2012	December 2015	Phase 3

**Colistin**	Usefulness of monitoring plasma levels of colistin and sodium colistimethate in patients with multidrug-resistant Gram-negative bacilli	January 2012	August 2015	Not applica ble
**Colistin**	Multicenter open-label randomized controlled trial to compare colistin alone *vs.* colistin + meropenem	March 2013	February 2017	Phase 4
**Colistin**	Colistin and rifampicin for multidrug-resistant *Acinetobacter*	November 2008	October 2011	Phase 3
**Colistin**	Colistin *vs.* colistin + fosfomycin for multidrug-resistant *Acinetobacter baumannii* infections	June 2010	May 2012	Phase 3
**Colistin**	Intravenous colistin for pulmonary exacerbations: improving safety and efficacy	August 2016	July 2021	Phase 4
**Colistin**	Effect of aerosolised colistin in ventilator associated pneumonia	April 2013	April 2015	Phase 4
**Colistin**	Intravenous colistin *vs.* intravenous colistin + nebulized colistin in ventilator-associated pneumonia due to multidrug-resistant *Acinetobacter baumannii*	April 2008	April 2011	Phase 3
**Colistin**	Effect of colistin inhalation on ventilator associated pneumonia	January 2016	January 2018	Phase 2/3
**Colistin**	Colistin + rifampicin in multidrug-resistant *P. aeruginosa* and *A. baumanii*	June 2010	August 2012	Phase 3
**Colistin**	Colistin pharmacokinetics in critically ill patients during extended dialysis	June 2013	May 2016	
**Colistin**	Renal impairment associated with colistin levels	December 2009	January 2011	
**Colistin**	Nebulized colistin for hospital-acquired pneumonia	June 2006		Phase 3
**Colistin**	Effect of different colistin doses on clinical outcome of pediatric cancer patients with Gram-negative bacteria infections	January 2017	June 2019	Phase 4
**Colistin**	Optimizing dosing of colistin for infections resistant to all other antibiotics	June 2008	August 2015	Not applica ble

**Colistin**	Sino-nasal inhalation of colistin in patients with cystic fibrosis and *Pseudomonas aeruginosa* colonization	December 2009	April 2012	Phase 2
**Colistin**	Aerosolized and intravenous colistin in healthy adults	August 2013	May 2017	Phase 1
**Colistin**	Impact of plasma levels of colistin in patients with carbapenem resistant *Acinetobacter baumannii* infection	May 2015	April 2018	
**Colistin**	Efficacy and safety of colistin for therapy of infections caused by extended spectrum-β-lactamase-producing *K. pneumoniae* or *E. coli*	February 2006	December 2006	Phase 3
**Colistin**	Nebulized and intravenous colistin in ventilator-associated pneumonia	July 2017	June 2018	Phase 3
**Colistin**	Incidence of infection after total knee arthroplasty using an erythromycin and colistin loaded cement or a standard cement	September 2007	May 2012	Not applicable
**Colistin**	Evaluation of pharmacokinetics and pharmacodynamics properties of intravenous colistimethate sodium	September 2013	June 2019	
**Colistin**	Investigate of the drug-drug interaction between multiple doses of RO7033877 and multiple doses of colistin methanesulfonate sodium	July 2014	March 2015	Phase 1
**Colistin**	Trial for the treatment of extensively drug-resistant Gram-negative bacilli	October 2012	September 2021	Phase 3
**Colistin**	Study of plazomicin compared with colistin in patients with infection due to carbapenem-resistant *Enterobacteriaceae*	September 2014	September 2016	Phase 3
**Colistin**	Study of colistin methanesulfonate sodium	October 2010	December 2010	Phase 1
**Colistin**	Eradication of antibiotic-resistant bacteria through antibiotics and fecal bacteriotherapy	February 2016	March 2018	Phase 2
**Colistin**	Efficacy, safety and tolerability of aztreonam-avibactam + metronidazole *vs.* meropenem + colistin for Gram-negative bacteria infections	April 2018	September 2021	Phase 3

**Colistin**	Clinical usefulness of therapeutic drug monitoring of colistin in treated patients	January 2016	December 2017	Not applica ble
**Colistin**	Selective digestive decontamination for eradicating carbapenem-resitant *Klebisella pneumonia* carriage	January 2013		Phase 4
**Colistin**	Comparison of 2 treatment regimens for eradication of *P. aeruginosa* infection in children with cystic fibrosis	August 2001	May 2011	Phase 4
**Colistin**	Microbiologic effect of selective decontamination of the digestive tract with colistin, gentamicin and nystatin			
**Colistin**	Impact of treatment with colistin on multiresistant *Acinetobacter baumannii* mortality, sensitive to colistin	January 2010		
**Colistin**	Population pharmacokinetics study of colistin in patients infected with multiresistant Gram-negative bacteria	May 2009		Phase 1
**Colistin**	Tracheobronchitis prevention trial	November 2009	May 2013	Not applica ble
**Colistin**	Selective digestive decontamination in carriers of carbapenem-resistant *Klebsiella pneumoniae*	November 2008	July 2010	Not applica ble
**Colistin**	Simplified selective digestive tract decontamination for the prevention of intensive care unit acquired infections	June 2013	July 2015	Phase 2/3
**Colistin**	Aztreonam for inhalation solution (AZLI) for the treatment of exacerbations of cystic fibrosis	November 2016	July 2019	Phase 4
**Colistin**	Assessment of kidney function for drug dosage adjustments in critically ill patients	February 2017	April 2019	Not applica ble

**Colistin**	Study to evaluate the efficacy and safety of intravenous sulbactam-ETX2514 in the treatment of patients with *Acinetobacter baumannii* infections	April 2019	July 2020	X	Phase 3
**Colistin**	Antibiotic administration in colorectal cancer surgery decrease in complications and better prognosis	January 2013	March 2017		Phase 4
**Colistin**	Decolonization in haematological malignancies	January 2017	December 2017		Phase 4
**Colistin**	Decontamination of the digestive tract in intensive care unit patients	May 2017	December 2021	X	Phase 3
**Colistin**	Scandinavian cystic fibrosis azithromycin study	May 2008	March 2014		Phase 4
**Colistin**	Antibiotic combination for infections caused by Gram-negative bacteria	January 2015	December 2015		Phase 2/3
**Colistin**	Drug monitoring of antibiotics in critical care patients	March 2013	January 2015		
**Colistin**	Control of intestinal colonization with fecal microbiota transplantation	January 2014	December 2016		Phase 2/3
**Colistin**	Pharmacokinetics of aerosolized colimycin in cystic fibrosis	October 2011			Phase 1
**Colistin**	Observational study of patients with drug resistant *Acinetobacter baumannii*	August 2010	June 2013		
**Colistin**	Efficacy of intestinal decontamination in patients colonized by carbapenem-resistant *Klebsiella pneumoniae* and colistin	July 2012	November 2015		
**Colistin**	7 *vs.* 14 days antibiotic therapy for ventilator-associated pneumonia	March 2018	May 2020	X	Not applica ble
**Colistin**	Evaluation of the role of oral antibiotics in carbapenem-resistant *Klebsiella pneumonia*	July 2009	January 2012		Not applica

					ble
**Colistin**	Cohort study on carbapenem-resistant *Klebsiella pneumonia*	June 2018	July 2019	X	
**Colistin**	Evaluation of inhaled antibiotics on bacterial diversity in cystic fibrosis lungs	April 2014	August 2016		
**Colistin**	Ecological effects of decolonization in intensive care	December 2013	October 2017		Phase 3
**Colistin**	Pharmacokinetics of antibiotics in renal replacement therapies patients	April 2018	July 2020	X	
**Colistin**	*Acinetobacter baumannii*-related osteomyelitis characterization	May 2017	December 2019	X	
**Colistin**	Impact of antimicrobials and MIC values on bloodstream infections due to extended-spectrum β-lactamase or carbapenemase-producing *Enterobacteriaceae*	January 2013	May 2014		
**Colistin**	Clinical outcomes and global epidemiology	July 2007	June 2017		
**Colistin**	Pharmacokinetics-pharmacodynamics study of 7 anti-infective agents in the cerebro spinal fluid of brain injured patients with an external ventricular drainage	July 2018	October 2020	X	Phase 1
**Colistin**	Ventilator-associated pneumoniadue to *Pseudomonas aeruginosa*	March 2018	July 2020	X	Phase 3
**Colistin**	Efficacy of colistimethate sodium inhalation in ventilator-associated pneumonia patients	October 2013	September 2016		
**Colistin**	Efficacy and safety of colistimethate sodium injection to hospital-acquired pneumonia	October 2013			Phase 2
**Colistin**	Efficacy and safety of imipenem + cilastatin/relebactam *vs.* colistimethate sodium + imipenem + cilastatin in imipenem-resistant bacterial infection	August 2015	September 2017		Phase 3
**Colistin**	Adherence improvement and clinical outcome in cystic fibrosis patients	December 2009	June 2011		Not applica ble
**Colistin**	Decolonization study of Enterobacteriaceae producing β-lactamases	June 2009	August 2012		Phase 4

**Colistin**	Expanded quantitative urinary culture *vs.* standard culture techniques in clinical care	June 2017	December 2019	X	Not applica ble
**Colistin**	Patient response to early switch to oral in osteomyelitis	April 2014	September 2019	X	Early Phase 1
**Colistin**	Efficacy, safety, and tolerability of aztreonam-avibactam in serious infection by metallo-β-lactamase producing Gram-negative bacteria treatment	April 2020	October 2021	X	Phase 3
**Colistin**	Treatment of non-cystic fibrosis bronchiectasis patients with chronic lung infections with colistimethate sodium	January 2018	July 2020	X	Phase 3
**Colistin**	Efficacy and safety of inhaled colistimethate sodium in bronchiectasis subjects with chronic *Pseudomonas aeruginosa* infection	June 2017	July 2020	X	Phase 3
**Colistin**	Gram-negative bacteremia in haematopoietic stem cell transplantation recipients	February 2014	April 2017		
**Colistin**	Cost of off-label antibiotics in osteoarticular infections	January 2017	April 2022	X	
**Colistin**	Usage of tobramycin inhalation powder *vs.* nebulised tobramycin inhalation solution and nebulised colistimethate	August 2013	October 2015		Phase 4
**Daptomycin**	Daptomycin as adjunctive therapy for *Staphylococcus aureus* bacteremia	November 2016	September 2019	X	Phase 4
**Daptomycin**	Daptomycin for antimicrobial prophylaxis in MRSA colonized patients undergoing hip, knee or shoulder arthroplasty	October 2010	March 2014		Phase 4
**Daptomycin**	Safety, efficacy, and pharmacokinetics of daptomycin in pediatric subjects with complicated skin and soft tissue infections and bacteremia	December 2018	May 2021		Phase 2
**Daptomycin**	Influence of rifampin co-administration on the pharmacokinetics profile of daptomycin	May 2014	July 2016		Early Phase 1
**Daptomycin**	The pharmacokinetics/pharmacodynamics of high-dose daptomycin in patients with septic	January	June 2016		Not

	shock	2015		applica ble
**Daptomycin**	Daptomycin in pediatric patients with bacterial meningitis	April 2012	June 2018	Phase 1
**Daptomycin**	Daptomycin with rifampin for treatment of staphylococcal prosthetic joint infection	June 2012	June 2016	Phase 2
**Daptomycin**	Pharmacokinetics variability of daptomycin during prolonged therapy for bone and joint infections	May 2015	September 2015	
**Daptomycin**	Vancomycin *vs.* daptomycin for the treatment of MRSA bacteremia	January 2014	December 2015	Phase 2
**Daptomycin**	Daptomycin pharmacokinetics in children with renal disease	November 2009	April 2014	Not applica ble
**Daptomycin**	Daptomycin concentration in drainage fluid and blood samples of intensive care unit patients	October 2016	March 2019	
**Daptomycin**	Daptomycin pharmacokinetics during cardiopulmonary bypass surgery	July 2008	October 2010	Phase 3
**Daptomycin**	Daptomycin pharmacokinetics in infants	January 2010	December 2011	Phase 1
**Daptomycin**	Daptomycin > 6 mg/kg/day as salvage therapy in patients with complex bone and joint infection	July 2014	January 2015	
**Daptomycin**	Cerebrospinal fluid pharmacokinetics of daptomycin	May 2010	May 2011	Not applica ble
**Daptomycin**	Daptomycin in treating neutropenia and fever in cancer patients	December 2006	October 2008	Phase 2
**Daptomycin**	Pharmacokinetics and safety evaluation of daptomycin in children between 2-6 years old with proven or suspected Gram-positive bacteria infections	June 2008	November 2008	Phase 1

**Daptomycin**	Efficacy and safety of daptomycin with and without combination therapy in *S. aureus* infective endocarditis	February 2009	November 2011		Phase 4
**Daptomycin**	Daptomycin pharmacokinetics in critically ill patients receiving continuous veno-venous hemodialysis	February 2007	April 2009		Phase 4
**Daptomycin**	Daptomycin as an adjuvant agent in the treatment of enterococcal native valve endocarditis	September 2006	October 2008		Phase 4
**Daptomycin**	Safety and efficacy of daptomycin for the treatment of complicated skin and skin-structure infections	January 2007			Phase 4
**Daptomycin**	Pharmacokinetics study of intraperitoneal administration of daptomycin in peritoneal infection	September 2013	December 2017		Not applica ble
**Daptomycin**	Retrospective analysis of nephrotoxicity with daptomycin *vs.* vancomycin treatments in high risk patients	January 2016	January 2016		
**Daptomycin**	Safety and efficacy of daptomycin *vs.* standard in 1-17 year olds with *Staphylococcus aureus* bacteremia	November 2012	January 2016		Phase 4
**Daptomycin**	Daptomycin in catheter-related *Staphylococcus aureus* treatment	May 2006	September 2012		Phase 2
**Daptomycin**	Application of daptomycin in MRSA infected diabetic foot in comparison to vancomycin treatment	July 2011	December 2013		Phase 3
**Daptomycin**	Daptomycin *vs.* vancomycin in nosocomial or healthcare-associated MRSA bacteremia treatment	May 2012	July 2014		Phase 3
**Daptomycin**	Daptomycin safety and pharmacokinetics in 3-24 months infants with proven/suspected Gram-positive bacteria infections or receiving prophylactic antibiotics	January 2010	March 2012		Phase 1
**Daptomycin**	Daptomycin *vs.* vancomycin in participants with skin infections due to MRSA	September 2011	October 2012		Phase 4
**Daptomycin**	Adjunction of daptomycin for pneumococcal meningitis treatment	June 2018	September 2021	X	Phase 2

**Daptomycin**	Evaluation of daptomycin given during dialysis and after dialysis	April 2009	July 2009	Phase 1
**Daptomycin**	Daptomycin as antibiotic prophylaxis of sternal wound infections	November 2008	March 2012	Phase 4
**Daptomycin**	Daptomycin *vs.* placebo in patients with neutropenia and fever	April 2011	April 2013	Phase 3
**Daptomycin**	Pharmacokinetics of daptomycin 10 mg/kg in patients with bacteremia, endocarditis or skin soft tissue infections due to Gram-positive bacteria with renal failure	March 2014		Phase 3
**Daptomycin**	Safety and efficacy of daptomycin in pediatric participants (1 to 17 years-old) with skin and skin structure infections	July 2008	October 2013	Phase 4
**Daptomycin**	Catheter-related Gram-positive bacteria bloodstream infections	March 2007		Phase 2
**Daptomycin**	Safety, efficacy, and pharmacokinetics of higher doses of daptomycin and vancomycin in MRSA bacteremia	September 2008	October 2010	Phase 2
**Daptomycin**	Daptomycin pharmacokinetics in continuous veno-venous hemodiafiltration	February 2010		Phase 2
**Daptomycin**	Daptomycin pharmacokinetics in healthy subjects	February 2009	March 2009	Phase 1
**Daptomycin**	Daptomycin for complicated skin and skin structure infections treatment	May 2012	July 2013	Phase 4
**Daptomycin**	Daptomycin for severe necrotizing soft-tissue infections treatment	June 2005	May 2008	Not applicable
**Daptomycin**	Efficacy and safety comparison of daptomycin in elderly patients with complicated skin and skin structure infections	March 2010	March 2011	Phase 3
**Daptomycin**	Safety evaluation of daptomycin in non-infected adults who are on hemodialysis or continuous ambulatory peritoneal dialysis	August 2007	May 2008	Phase 1
**Daptomycin**	Efficacy of daptomycin + fosfomycin *vs.* daptomycin for MRSA bacteremia treatment	December 2013	January 2018	Phase 3
**Daptomycin**	Daptomycin for renal insufficiency and complicated skin and skin structure infections treatment	January	November	Phase 4

		2005	2006		
**Daptomycin**	Daptomycin safety and efficacy compared to active comparator in pediatric participants with acute hematogenous osteomyelitis	September 2013	December 2016		Phase 3
**Daptomycin**	Daptomycin + meropenem *vs.* ceftazidime in the treatment of nosocomial spontaneous bacterial peritonitis	October 2010	July 2014		Phase2/3
**Daptomycin**	Real-world daptomycin use in Chinese intensive care units	June 2018	December 2021	X	
**Daptomycin**	Cubicin comparison with vancomycin in complicated skin and skin structure infections in a home infusion setting treatment	July 2010	November 2011		Phase 4
**Daptomycin**	Daptomycin for antimicrobial prophylaxis	January 2008	September 2008		Phase 4
**Daptomycin**	Cubicin for complicated post-surgical wound infections	June 2004	March 2005		Phase 4
**Daptomycin**	Daptomycin safety and efficacy *vs.* vancomycin or teicoplanin for complicated skin and skin structure infections treatment	April 2006			Phase 3
**Daptomycin**	Daptomycin for infective endocarditis or to *S. aureus* bacteremia treatment	March 2002	February 2005		Phase 3
**Daptomycin**	Study comparing CUBICIN® (daptomycin for injection) with vancomycin in cellulitis or erysipelas	February 2006	August 2006		Phase 4
**Daptomycin**	Collection of daptomycin pharmacokinetics samples in patients with renal failure	March 2008	December 2010		
**Daptomycin**	Daptomycin and cytokines production effects in comparison with vancomycin	July 2012	March 2015		Phase 4
**Daptomycin**	Daptomycin safety and efficacy for complicated skin and skin structure infections and bacteremia in renal impairment	April 2010	June 2012		Phase 4
**Daptomycin**	Vancomycin and daptomycin + cefazolin for preoperative vascular surgery prophylaxis	August 2007	August 2010		Phase 4
**Daptomycin**	Outpatient treatment of Gram-positive bacteria wound infections in diabetic foot comparing	August	August		Phase 4

	daptomycin *vs.* vancomycin based regimens	2010	2012		
**Daptomycin**	Comparative study of daptomycin to ceftriaxone in moderate to severe community-acquired acute bacterial pneumonia treatment	October 2000	September 2001		Phase 3
**Daptomycin**	Single-dose daptomycin in pediatric patients with Gram-positive bacteria infection for which they are receiving standard antibiotics	August 2005	August 2006		Phase 1
**Daptomycin**	Daptomycin for Gram-positive bacteria infections treatment	December 2002	January 2004		Phase 3
**Daptomycin**	Efficacy and safety study of brilacidin to treat serious skin infections	February 2014	October 2014		Phase 2
**Daptomycin**	Clinical evaluation of daptomycin bone penetration	May 2011	November 2015		
**Daptomycin**	Daptomycin pharmacokinetics in critically ill patients undergoing continuous veno-venous hemodiafiltration	December 2009	March 2010		Phase 2
**Daptomycin**	Daptomycin in subjects undergoing surgery for osteomyelitis associated with an infected prosthetic caused by Staphylococci	June 2007	June 2010		Phase 2
**Daptomycin**	Population pharmacokinetics analysis of daptomycin in patients with osteoarticular infections	December 2016	June 2017		
**Daptomycin**	China registration study in patients with skin infections	September 2008	September 2010		Phase 3
**Daptomycin**	Retrospective evaluation of safety and efficacy of daptomycin used in patients with serious Gram-positive bacteria infections	February 2011	April 2011		
**Daptomycin**	Safety and efficacy study of daptomycin in complicated skin and skin structure infections	January 2007	September 2007		Phase 2
**Daptomycin**	Vancomycin *vs.* daptomycin for the treatment of MRSA bacteremia due to isolates with high vancomycin MICs	June 2011	January 2012		Phase 4
**Daptomycin**	Ceftobiprole in the treatment of patients with *Staphylococcus aureus* bacteremia	June 2018	August	X	Phase 3

			2021		
**Daptomycin**	Study of daptomycin to ceftriaxone in the treatment of moderate to severe community-acquired acute bacterial pneumonia due to *S. pneumoniae*	July 2001	February 2002		Phase 3
**Daptomycin**	IL-10 stratifying tool towards antibiotic selection for MRSA	November 2015	January 2018		Phase 4
**Daptomycin**	Clinical data collection in Chinese patients received CUBICIN treatment for actual usage in clinical practice	January 2011	November 2012		
**Daptomycin**	Study of MK-3009 in Japanese patients with skin or blood stream infections caused by MRSA	September 2008	February 2010		Phase 3
**Daptomycin**	Initial treatment for acute bacterial skin infections caused by *Staphylococcus aureus*	October 2010	March 2012		Phase 2
**Daptomycin**	Vascular surgical antibiotic prophylaxis study	May 2011	October 2013		Phase 4
**Daptomycin**	Safety and efficacy of ceftaroline in subjects with *Staphylococcus aureus* bacteremia or with persistent MRSA bacteremia	January 2013	July 2014		Phase 4
**Daptomycin**	Telavancin *Staphylococcus aureus* bacteremia trial	December 2014	April 2018		Phase 3
**Daptomycin**	Retrospective evaluation of safety and efficacy of daptomycin used in patients with serious Gram-positive bacteria infections	February 2011			
**Daptomycin**	Concentration of antimicrobials in catheter-lock solutions	May 2012	December 2015		Phase 4
**Daptomycin**	Antibiotic plasma concentrations during continuous renal replacement therapy with a high adsorption membrane	August 2019	December 2022	X	
**Daptomycin**	Combinational antibiotic therapy for MRSA infection	August 2015	October 2018		Phase 3
**Daptomycin**	Safety and efficacy of drug-induced nephrotoxicity in high-risk patients prevention	October			Phase 4

		2011			
**Daptomycin**	Dalbavancin for Gram-positive bacteria osteoarticular infections treatment	January 2018	January 2021	X	Phase 4
**Daptomycin**	Acute renal failure in surgical intensive care units	July 2006	December 2012		Not applicable
**Daptomycin**	*Staphylococcus aureus* bacteremia antibiotic treatment options	December 2013	October 2019	X	Phase 3
**Daptomycin**	Study of MRSA isolated from infected patients in Brazil	July 2011			
**Daptomycin**	Efficacy of oral antibiotic therapy compared to intravenous antibiotic therapy for osteomyelitis	March 2014	February 2017		Phase 2
**Daptomycin**	Off-label antibiotics cost in osteoarticular infections	January 2017	April 2022	X	
**Daptomycin**	Infection control in high risk patients of developing *Clostridium difficile* infection, vancomycin-resistant enterococci or multidrug-resistant Gram-negatives	November 2016	December 2020	X	
**DPK-060**	Clinical safety and efficacy of DPK-060 in acute external otitis patients	November 2011	November 2012		Phase 2
**DPK-060**	Investigation of the antimicrobial efficacy and safety of DPK-060 topical application	March 2008	April 2009		Phase1/2
**Friulimicin B**	Friulimicin B human trial	June 2007	September 2007		Phase 1
**Friulimicin B**	Pharmacokinetics/pharmacodynamics of EA-230 during endotoxemia	February 2015	December 2015		Phase 1
**Friulimicin B**	Immunomodulation of EA-230 following on-pump coronary artery bypass grafting	June 2016	October 2018		Phase 2
**hLF-1**	Safety of a single dose of hLF1-11 to autologous haematopoietic stem cell transplant recipients	March 2006	November		Phase

			2006		1/ 2
**Iseganan**	Iseganan in ventilator-associated pneumonia prevention	September 2003	June 2004		Phase2/ 3
**Iseganan**	Iseganan hydrochloride in oral mucositis prevention in patients undergoing radiation therapy for head and neck cancer	December 2000			Phase 3
**LL-37**	Intratumoral injection of LL-37 for melanoma	July 2015	July 2021	X	Phase 1/2
**LL-37**	Effect of smoking and vitamin D_3 on human cathelicidin peptide LL-37 levels	December 2011	July 2015		
**LL-37**	Passive smoking and LL-37 in children	November 2016	June 2018		
**LL-37**	Evaluation of the inhibitory effects of topical ivermectin on markers of Rosacea-specific inflammation	July 2016	August 2019		Phase 1/2
**LL-37**	High dose preoperative cholecalciferol supplementation and perioperative vitamin D status	January 2013	May 2015		Phase 3
**LL-37**	Effects of aminocaproic acid on Rosacea-specific inflammation	July 2011	December 2012		Early Phase 1
**LL-37**	Effect of high-dose vitamin D_3 in smokers and non-smokers with and without HIV	April 2018	June 2020	X	Phase 1
**LL-37**	Comparison of cathelicidin expression in skin and saliva in atopic dermatitis and psoriasis patients	July 2005	February 2009		
**LL-37**	Therapeutic induction of endogenous antibiotics	January 2005	January 2009		Phase 2
**LL-37**	Phenylbutyrate and vitamin D in tuberculosis	December 2010	December 2014		Phase 2
**LL-37**	Effects of surfactants on the innate immune system	September 2013	December 2015		Not applica

					ble
**LL-37**	Calcitriol *vs.* placebo among critically-ill patients with sepsis	February 2013	February 2014		Phase 2
**LL-37**	Effects of vitamin D supplementation on lung function in cystic fibrosis	October 2008	September 2010		Not applica ble
**LL-37**	Effects of vitamin D and omega-3 lipids on infectious diseases and hCAP18	July 2010	December 2019	X	Not applica ble
**LL-37**	Cholecalciferol supplementation for sepsis in the intensive care unit	January 2014	December 2014		Phase 3
**LL-37**	Fermented milk on the appearance of common winter infectious diseases	December 2014	June 2015		Phase 2
**LL-37**	Exploratory pharmacodynamics study of HP802-247 in venous leg ulcers	July 2014	October 2015		Phase 2
**LL-37**	cAMP (cyclic adenosine monophosphate) levels in the nose	March 2011	December 2012		Not applica ble
**LL-37**	Vitamin D in ventilated intensive care unit patients	July 2011	April 2014		Phase 2
**LL-37**	Effects of *Lactobacillus reuteri* and vitamin D_3 in children with atopic dermatitis	February 2015	February 2018		Not applica ble
**LL-37**	Immune reconstitution in HIV disease	September 2012	August 2015		Phase 2
**LL-37**	Immune reconstitution in tuberculosis disease	September 2012	August 2015		Phase 2

**LL-37**	Evaluation of rosacea-related inflammatory markers in adult skin when treated with LL-37 *vs.* placebo	April 2011	August 2012		Phase 4
**LL-37**	Evaluation of bifidobacterium BB-12® efficacy in the treatment of infantile colic	November 2016	November 2017		Not applica ble
**LL-37**	Safety, tolerability, pharmacokinetics, pharmacodynamics and food effect of single or repeat doses of GSK2793660 in healthy subjects	January 2014	October 2014		Phase 1
**LL-37**	Effects of Antarctic environment on vitamin D status and health risk biomarkers	October 2016	March 2018		Not applica ble
**LL-37**	Antenatal vitamin D_3 dose-finding and safety study	July 2009	June 2010		Phase 1
**LL-37**	Etiology of eczema herpeticum	February 2017	June 2020	X	
**LL-37**	Cellular immunotherapy for septic shock	March 2018	October 2020	X	Phase 2
**LL-37**	Effects of vitamin D supplementation on aerobic fitness in athletes	October 2014	April 2015		Phase 4
**LL-37**	Efficacy of vitamin D supplementation to MRSA carriers	December 2014	December 2017		Phase 2
**LTX-109**	Efficacy and safety of two doses of LTX-109 in impetigo	February 2013	April 2014		Phase 2
**LTX-109**	Safety, tolerability and efficacy of topical LTX-109 in nasal MRSA/MSSA	May 2010	April 2011		Phase 1/2
**LTX-109**	Safety, tolerability and efficacy of LTX-109 on uncomplicated Gram-positive bacteria skin infection	September 2010	February 2011		Phase 2
**MSI-78**	MSI-78 for topical application *vs.* oral ofloxacin in infected diabetic ulcers treatment	August	July 1996		Phase 3

		1994			
**MSI-78**	MSI-78 for topical application *vs.* oral ofloxacin in infected diabetic ulcers treatment	August 1994	July 1996		Phase 3
**Nisin**	Inhibition effects of probiotics on pathogens associated with ventilator-associated pneumonia	November 2016	January 2018		
**Nisin**	Functional ingredients: effect in gastrointestinal system	April 2012	December 2012		Not applica ble
**Omiganan**	Twice a day omiganan in facial seborrheic dermatitis patients	October 2018	December 2019	X	Phase 2
**Omiganan**	Evaluation of long-term safety of a once-daily omiganan topical gel	September 2015	February 2018		Phase 3
**Omiganan**	Antimicrobial activity of omiganan 1% gel *vs.* chlorhexidine 2% for topical skin antisepsis in healthy adult subjects	May 2008	June 2008		Phase 3
**Omiganan**	Pharmacodynamics of omiganan BID in patients with atopic dermatitis	March 2017	December 2017		Phase 2
**Omiganan**	Pharmacodynamics, safety and efficacy of topical omiganan in patients with external genital warts	July 2016	March 2017		Phase 2
**Omiganan**	Pharmacodynamics, safety/tolerability and efficacy of topical omiganan in patients with vulvar intraepithelial neoplasia	November 2015	July 2017		Phase 2
**Omiganan**	Omiganan 1% gel preventing catheter infections/colonization in patients with central venous catheters	August 2005	July 2008		Phase 3
**Omiganan**	Topical challenge with omiganan and imiquimod in healthy volunteers	February 2017	March 2017		Phase 1
**Omiganan**	Safety and efficacy evaluation of omiganan topical gel *vs.* vehicle in female subjects with moderate to severe acne vulgaris	October 2015	August 2016		Phase 2

**Omiganan**	Pharmacodynamics of omiganan in atopic dermatitis patients	May 2015	November 2015	Phase 2
**Omiganan**	A twelve week safety and efficacy study in Rosacea	March 2013		Phase 2
**Omiganan**	Safety and efficacy evaluation of a once-daily topical gel *vs.* vehicle	October 2015	April 2018	Phase 3
**Omiganan**	Safety and efficacy evaluation of a once-daily topical gel *vs.* vehicle	October 2015	September 2017	Phase 3
**Omiganan**	Safety and efficacy of MBI 226 for central venous catheter-related bloodstream infections prevention	September 2000	July 2003	Phase 3
**Omiganan**	Safety and efficacy of MBI 226 1.25% and 2.5% topical acne solutions in acne treatment	January 2003	September 2003	Phase 2
**Omiganan**	Safety and efficacy of MBI 226 1.25% and 5% topical acne solutions in acne treatment	October 2000	September 2001	Phase 2
**PAC113**	PAC113 mouthrinse in HIV seropositive individuals with oral candidiasis	January 2008	June 2008	Phase 2
**Pexiganan**	Pexiganan *vs.* placebo for mild infections of diabetic foot ulcers treatment	June 2014	July 2016	Phase 3
**Pexiganan**	Pexiganan *vs.* placebo control for the treatment of mild infections of diabetic foot ulcers	June 2014	August 2016	Phase 3
**POL7080**	POL7080 pharmacokinetics, safety and efficacy in ventilator-associated *Pseudomonas aeruginosa* pneumonia patients	October 2013	December 2016	Phase 2
**POL7080**	POL7080 pharmacokinetics / pharmakodynamics, safety and efficacy in exacerbation of non-cystic fibrosis bronchiectasis patients	December 2013	November 2015	Phase 2
**POL7080**	POL7080 pharmacokinetics and safety in renal impairment patients	April 2013	July 2015	Phase 1
**POL7080**	Interaction between amikacin and POL7080	April 2016	December 2016	Phase 1
**POL7080**	Nosocomial pneumonia suspected or confirmed due to *Pseudomonas*	October	December	Phase 3

		2018	2021		
**POL7080**	Suspected or confirmed ventilator-associated pneumonia due to *Pseudomonas aeruginosa*	March 2018	July 2020		Phase 3
**Polymyxin B**	Efficacy of polymyxin B against infections caused by extensively drug-resistant Gram-negative bacteria	December 2014	November 2015		Phase 4
**Polymyxin B**	Polymyxin B monotherapy *vs.* combination in critically ill patients with multidrug-resistant pathogens	May 2017	December 2019	X	Phase 3
**Polymyxin B**	Melatonin for renal protection in patients receiving polymyxin B	October 2018	July 2020	X	Phase 2
**Polymyxin B**	Optimizing clinical use of polymyxin B	February 2016	May 2020	X	
**Polymyxin B**	Impact of early peri-operative use of polymyxin B hemoperfusion in septic patients undergoing emergent abdominal surgery	January 2012	February 2015		Not applicable
**Polymyxin B**	The effects of polymyxin B protects on sepsis induced kidney dysfunction: a randomized clinical trial	May 2006	December 2007		Phase 3
**Polymyxin B**	Safety and efficacy of heparin dosing score protocol for polymyxin B hemoperfusion	May 2017	July 2017		
**Polymyxin B**	The pilot study of the efficacy of polymyxin B hemoperfusion in critically ill patients with severe sepsis	January 2015	August 2017		Not applicable
**Polymyxin B**	Efficacy of polymyxin B hemoperfusion on severe sepsis secondary to pneumonia or urinary tract infection	August 2016	March 2019		Not applicable
**Polymyxin B**	Randomized-controlled trial on combination antibiotic for infections caused by Gram-negative bacteria	January 2015	December 2015		Phase 2/3
**Polymyxin B**	Effectiveness of polymyxin B sulphate + prednisolone + benzocaine + clioquinol in acute and sub-acute dermatitis eczematous	May 2012	August 2012		Phase 3

**Polymyxin B**	Polyethyleneimine-coated membrane for hemofiltration *vs.* polymyxin B-immobilized fibre column in septic shock	August 2013	July 2019		Not applica ble
**Polymyxin B**	Early use of polymyxin B hemoperfusion in abdominal sepsis	December 2004	April 2008		Phase 4
**Polymyxin B**	Open-label evaluation of polymyxin B hemoperfusion for septic shock				
**Polymyxin B**	Effects of hemoperfusion with a polymyxin B membrane in peritonitis with septic shock	October 2010	December 2013		Phase 3
**Polymyxin B**	Safety and efficacy of polymyxin B hemoperfusion for septic shock	June 2010	June 2017		Not applica ble
**Polymyxin B**	Gut decontamination in pediatric allogeneic hematopoietic stem cell transplantation	March 2016	May 2023		Phase 2
**Polymyxin B**	Antibiotic irrigation for pancreatoduodenectomy	November 2014	August 2024	X	Not applica ble
**Polymyxin B**	Efficacy Maxinom® and Maxitrol® in reducing the signs and symptoms of acute bacterial conjunctivitis	February 2011			Phase 3
**Polymyxin B**	Comparison of combination antibiotics eyedrop to artificial tear in hordeolum after incision and curettage	September 2007	December 2008		Phase 3
**Polymyxin B**	Endotoxin adsorber hemoperfusion and microcirculation	December 2012	June 2019		Not applica ble
**Polymyxin B**	Safety and efficacy of polymyxin B hemoperfusion for endotoxemic septic shock	July 2019	December 2021		Not applica ble
**Polymyxin B**	Clearance of nasal *Staphylococcus aureus* with triple antibiotic ointment	September	November		Not

		2009	2010		applica ble
**Polymyxin B**	Safety evaluation and immunogenicity of monovalent oral polio vaccine type 2 in children aged 1 to 5 years in Lithuania	November 2015	December 2016		Phase 4
**Polymyxin B**	The combined antibiotic therapy for carbapenem resistant *Klebsiella pneumoniae*	January 2019	December 2020	X	Early Phase 1
**Polymyxin B**	Treatment of acyclovir-resistant mucocutaneous *Herpes simplex* disease in patients with AIDS		April 1992		Not applica ble
**Polymyxin B**	Efficacy of POLYGYNAX® in the empirical treatment of infectious vaginitis	September 2015	August 2016		Phase 3
**Polymyxin B**	Safety and efficacy study of polymyxin B for congenital nasolacrimal duct obstruction in children treatment	September 2011	June 2014		Phase 1
**Polymyxin B**	Ear comfort ear drops to treat acute otitis externa cases compared to DexOtic® and Otidin®	July 2011	December 2013		Phase 2
**Polymyxin B**	Effect of systemic oxygen on corneal epithelial wound healing in diabetic patients undergoing pars plana vitrectomy	October 2013	October 2014		Not applica ble
**Polymyxin B**	The effect of glaucoma drops on the duration of a pure intraocular gas bubble	June 2011	April 2013		Not applica ble
**Polymyxin B**	Efficacy and safety study of CA330 hemoadsorption device on IL-6 removal in septic patients	December 2018	October 2020	X	Not applica ble
**Polymyxin B**	Moxifloxacin *vs.* Polytrim® for conjunctivitis	March 2007	March 2012		Phase 4
**Polymyxin B**	Intravitreal carboplatin for the treatment of participants with recurrent or refractory intraocular	November	December	X	Phase 1

	retinoblastoma	2016	2024		
**Polymyxin B**	Comparison between bandage contact lens and pressure patching for the erosion area and pain scale in corneal erosion patients	September 2012	February 2013		Phase 3
**Polymyxin B**	Perioperative antibiotic therapy to prevent cardiac implantable electronic device infections	April 2016	August 2020	X	Phase 3
**Polymyxin B**	Alcohol 20% for separation of pterygium and comparison of different wound closure methods	June 2008	July 2010		Not applica ble
**Polymyxin B**	Frozen *vs.* fresh corneal carriers for the Boston KPro type I donor carriers	September 2013	January 2020	X	Not applica ble
**Polymyxin B**	Efficacy, safety and tolerability of aztreonam-avibactam in the treatment of serious infection due to metallo-β-lactamase-producing Gram-negative bacteria	April 2020	October 2021	X	Phase 3
**Polymyxin B**	Study of subretinally injected SAR439483 administered in patients with Leber congenital amaurosis caused by biallelic mutations in GUCY2D	July 2019	December 2021	X	Phase 1/2
**Polymyxin B**	Biafine cream *vs.* standard care in subjects with actinic keratosis post cryotherapy	October 2006	February 2008		Phase 4
**Polymyxin B**	Patients response to early switch to oral in osteomyelitis	April 2014	September 2019	X	Early Phase 1
**Polymyxin B**	Use of ointments in prevention of catheter related infections in peritoneal dialysis	February 2006	June 2010		Phase 4
**Polymyxin B**	Tacrolimus for malar edema	October 2018	July 2019		Phase2/ 3
**Polymyxin B**	Effect of nitroglycerin ointment on mastectomy flap necrosis	August 2012	June 2014		Not applica ble

**Polymyxin B**	Cytoscopic antibiotic irrigant to reduce postoperative urinary tract infection	April 2016	June 2019		Phase 4
**Polymyxin B**	Two trabecular micro-bypass stents and postoperative travoprost to treat glaucoma subjects on two hypotensive agents	March 2013	April 2019		Phase 4
**Polymyxin B**	Photo-activated disinfection *vs.* triple-antibiotic paste for management of necrotic young permanent anterior teeth	October 2016	April 2019		Not applica ble
**Polymyxin B**	Comparison of Mepitel® Ag *vs.* antibiotic ointment used with soft cast technique for treatment of pediatric burns	September 2016	December 2019	X	Phase 4
**Polymyxin B**	Tissue characterization in teeth treated with a regeneration protocol	March 2010	March 2014		Early Phase 1
**Polymyxin B**	Next science wound gel efficacy in chronic wound *vs.* standard of care	July 2014	September 2016		Not applica ble
**Polymyxin B**	Antibiotic ointments and cream sting after application on a minor wound after tape stripping injury	March 2015	March 2015		Not applica ble
**Polymyxin B**	3Mixstatin, tri-antibiotic mix and simvastatin in the treatment of primary molars	September 2018	January 2020	X	Not applica ble
**Polymyxin B**	Effect of triple antibiotic paste as an intracanal medication with an anti-inflammatory drug on post-operative pain of uniradicular necrotic teeth	October 2016	January 2018		Not applica ble
**Polymyxin B**	Suppression of postprandial monocyte activation by blueberries or docosahexaenoic acid in humans	June 2015	November 2017		Not applica ble
**Polymyxin B**	Triple antibiotic paste, ciprofloxacin/propolis, ciprofloxacin/metronidazole,	January	January		Phase 4

	propolis/metronidazole on revascularization process of necrotic maxillary incisors	2015	2018		
**Polymyxin B**	Treatment outcome with a revascularization protocol using the triple *vs.* double antibiotic paste	April 2009	January 2015		Early Phase 1
**Polymyxin B**	Surgical site infection with 0.05% chlorhexidine compared to triple antibiotic irrigation	December 2015	December 2022	X	Not applica ble
**Polymyxin B**	Regeneration of pulp-dentin development in teeth with necrotic pulps and immature roots	September 2014	December 2019	X	Phase 1
**Polymyxin B**	Research of the application of triple antibiotic paste in primary teeth with pericpical periodontitis	June 2013	June 2015		Not applica ble
**PXL01**	PXL01 *vs.* placebo to inhibit adhesion formation after flexor tendon surgery	December 2009	February 2013		Phase 2
**PXL01**	Local tolerability safety and pharmacokinetics of PXL01 in healthy volunteers	March 2009	April 2009		Phase1
**SGX942**	SGX942 for oral mucositis treatment in patients with concomitant chemoradiation therapy for head and neck cancer	December 2017	December 2020	X	Phase 3
**SGX942**	Dose-escalating study of SGX942 for oral mucositis in patients with head and neck cancer	December 2013	September 2016		Phase 2
**Surotomycin**	Multiple doses of oral surotomycin in healthy participants	July 2009	October 2009		phase 1
**Surotomycin**	Single doses of oral surotomycin in healthy participants	January 2009	March 2009		Phase 1
**Surotomycin**	CB-183,315 in participants with *Clostridium difficile a*ssociated diarrhea	May 2012	March 2015		Phase 3
**Surotomycin**	CB-183,315 in participants with *Clostridium difficile* associated diarrhea	May 2012	August 2015		Phase 3
